# Prevalence of Overweight and Obesity in People With Severe Mental Illness: Systematic Review and Meta-Analysis

**DOI:** 10.3389/fendo.2021.769309

**Published:** 2021-11-25

**Authors:** Medhia Afzal, Najma Siddiqi, Bilal Ahmad, Nida Afsheen, Faiza Aslam, Ayaz Ali, Rubab Ayesha, Maria Bryant, Richard Holt, Humaira Khalid, Kousar Ishaq, Kamrun Nahar Koly, Sukanya Rajan, Jobaida Saba, Nilesh Tirbhowan, Gerardo A. Zavala

**Affiliations:** ^1^ Department of Health Sciences, University of York, York, United Kingdom; ^2^ Hull York Medical School, York, United Kingdom; ^3^ Health and Care of Older People, East Kent Hospitals University National Health Service (NHS Foundation), Kent, United Kingdom; ^4^ Institute of Psychiatry, Rawalpindi Medical University, Rawalpindi, Pakistan; ^5^ Human Development and Health, Faculty of Medicine, University of Southampton, Southampton, United Kingdom; ^6^ Southampton National Institute for Health Research Biomedical Research Centre, University Hospital Southampton NHS Foundation Trust, Southampton, United Kingdom; ^7^ Health System and Population Studies Division, International Centre for Diarrhoeal Disease Research, Dhaka, Bangladesh; ^8^ Department of Psychiatry, National Institute of Mental Health and Neurosciences, Bangalore, Bangalore, India

**Keywords:** severe mental illness (SMI), schizophrenia, bipolar disorder, obesity, overweight, systematic review & meta-analysis

## Abstract

**Aims:**

1) To determine the pooled prevalence of overweight and obesity in people with severe mental illness (SMI), overall and by type of SMI, geographical region, and year of data collection; and 2) to assess the likelihood of overweight and obesity, in people with SMI compared with the general population.

**Methods:**

PubMed, Medline, EMBASE, and PsycINFO databases were searched to identify observational studies assessing the prevalence of obesity in adults with SMI. Screening, data extraction and risk of bias assessments were performed independently by two co-authors. Random effect estimates for the pooled prevalence of overweight and obesity and the pooled odds of obesity in people with SMI compared with the general population were calculated. Subgroup analyses were conducted for types of SMI, setting, antipsychotic medication, region of the world, country income classification, date of data collection and sex. We assessed publication bias and performed a series of sensitivity analyses, excluding studies with high risk of bias, with low sample size and those not reporting obesity according to WHO classification.

**Result:**

120 studies from 43 countries were included, the majority were from high income countries. The pooled prevalence of obesity in people with SMI was 25.9% (95% C.I. = 23.3-29.1) and the combined pooled prevalence of overweight and obesity was 60.1% (95% C.I. = 55.8-63.1). Sub-Saharan Africa (13.0%, 95%C.I. = 6.7-25.1) and South Asia (17.7%, 95%C.I. = 10.5-28.5) had the lowest prevalence of obesity whilst North Africa and the Middle East (35.8%, 95%C.I. = 23.8-44.8) reported the highest prevalence. People with SMI were 3.04 more likely (95% C.I. = 2.42-3.82) to have obesity than the general population, but there was no difference in the prevalence of overweight. Women with schizophrenia were 1.44 (95% C.I. = 1.25-1.67) times more likely than men with schizophrenia to live with obesity; however, no gender differences were found among those with bipolar disorder.

**Conclusion:**

People with SMI have a markedly high prevalence and higher odds of obesity than the general population. This may contribute to the very high prevalence of physical health conditions and mortality in this group. People with SMI around the world would likely benefit from interventions to reduce and prevent obesity.

## Introduction

Obesity is a major global public health challenge (1). The prevalence has steadily risen over recent decades, with rates tripling since 1975 to the point where 30% of the world’s population has either overweight or living with obesity (1). According to the World Health Organization (WHO), the body mass index (BMI) provides the most useful, albeit crude, measure of population-level obesity (2). People with severe mental illness (SMI), (defined as those with schizophrenia and psychotic disorders, bipolar disorder and major depressive disorder with psychotic features), have been reported to be disproportionately affected by the obesity epidemic (3, 4). Studies from different countries and settings (inpatient, outpatient and community) around the world have reported prevalence of obesity ranging from 10% to 60%, suggesting the risk and prevalence of obesity in this population might vary by setting, country and region of the world (5, 6). Current data suggest that the prevalence of obesity among people with SMI is increasing at a faster rate than the general population (7). The high prevalence of obesity has been attributed to a combination of side effects and increased appetite associated with psychotropic medication, clustering of health risk behaviours such as poor diet with a high content of saturated fats (8), low engagement with physical activity (9), and social determinants of poor health such as stigma and poverty (10).

Obesity leads to metabolic disorders such as diabetes and cardiovascular disease, musculoskeletal disorders, cancers and premature death. People with SMI have nearly twice the risk of dying from cardiovascular disease and die on average 10 to 20 years earlier than the general population (11). Concerningly, this mortality gap appears to have widened in recent decades (12, 13). It is estimated that 33% of the excess mortality in SMI is attributable to obesity and its comorbidities (14–16).

Currently there are no systematic reviews reporting the prevalence and trends of obesity in people with SMI. Previous reviews have focused predominantly on the metabolic syndrome in this population (11, 12, 17). One review included data for all types of SMI and two reviews focused solely on people with schizophrenia. In all these studies, only abdominal obesity based on waist circumference was reported as a component of metabolic syndrome or as a secondary outcome, which means that studies that measured obesity using body mass index (BMI) may have been missed. This is an important gap as overweight and obesity are found to be earlier on the causal pathway that leads to metabolic syndrome, and thus deserve to be explored independently (9). Moreover, separately from the metabolic pathway, they are associated with additional adverse outcomes (e.g. osteoarthritis, mental disorders and sleep apnoea).

An up-to-date synthesis examining the prevalence of overweight and obesity is needed to raise awareness amongst stakeholders and policymakers about the extent of the obesity epidemic in people with SMI. This can inform strategies and interventions to address the problem and help reduce health inequalities for this population. The aims of this study were: 1) to determine the overall pooled prevalence of overweight and obesity and prevalence by type of SMI, setting, geographical region, country economy classification, year of data collection and sex; and 2) to assess the likelihood of obesity in people with SMI compared with matched controls from the general population.

## Materials and Methods

This systematic review was conducted in accordance with the Preferred Reporting Items for Systematic Reviews and Meta-Analyses (PRISMA) (18) and Meta-analysis Of Observational Studies in Epidemiology (MOOSE) guidelines (19). The protocol has been registered and published in the International prospective register of systematic reviews (PROSPERO) CRD-42020200380 (20).

### Search Strategy

We conducted a systematic search of PubMed, Medline, EMBASE, and PsycINFO databases with the help of an information specialist. Studies published from the date of inception up to July 2020 were included. Combining the following key words, the search terms used were: 1) Population (“severe mental illness” OR “serious mental illness” OR “schizophrenia” OR “psychosis” OR “psychotic” OR “psychotic disorder*” OR “psychosis” OR “schizoaffective” OR “schizo-affective” OR “bipolar disorder*” OR “mania” OR “manic” OR “bipolar” OR “depression” OR “major depressive disorder” or “antipsychotic”); 2) Outcome ***(*** BMI OR “Body Mass Index” OR weight OR “waist circumference” OR “waist to hip ratio” OR “percentage body fat” OR obesity OR overweight OR underweight OR thinness OR undernourished* OR malnutrition * OR undernutrition * OR adiposity); 3) study design **(** epidemiology* OR prevalence OR cross-sectional OR “ cohort” OR longitudinal OR observant OR observational OR case-control OR survey). In addition, we conducted citation searches for included studies and relevant systematic reviews to identify any relevant additional studies.

### Study Selection

For the systematic review and the meta-analysis we included studies according to the following criteria:

1) Studies including adult populations (aged ≥18 years) with a diagnosis of a SMI (schizophrenia spectrum disorders, bipolar affective disorder and major depression with psychosis), using definitions of any standardised tool such as the International Classification of Diseases (ICD) (3), or the Diagnostic and Statistical Manual of Mental Disorders (DSM) (21);
2) Studies reporting the prevalence of obesity using body mass index (BMI) using any established standardised definition, such as the WHO (22, 23);
3) Observational studies (e.g., cross-sectional, prospective or retrospective cohort studies, and case-control or studies).

Qualitative studies, studies with a sample size below 30, case reports, and studies where the majority of the population were under 18 years of age were excluded. We originally intended to include papers in any language, however due to time and resource restrictions, we excluded papers in a language other than English.

### Outcomes

Primary: prevalence of obesity among people with SMI. Secondary: Prevalence of overweight and combined prevalence of overweight and obesity among people with SMI; and prevalence of overweight, obesity and combined prevalence of overweight and obesity in matched controls from the general population (when available). Overweight (25-29.9 Kg/m^2^) and obesity (≥30 Kg/m^2^) were defined according to the WHO BMI cut-off points for most populations (22). However, we also included studies presenting ethnicity adjusted classifications which usually have a lower cutoff score for overweight (23–27.4 kg/m^2^) and obesity (≥27.5 Kg/m^2^) (23).

### Data Extraction (Selection and Coding)

Data screening and selection were completed using Covidence (Melbourne, Australia) (24). Two of four independent co-authors (AA, HK, MA, NT) completed screening of records (titles and abstracts); disagreements were resolved by consensus or in consultation with a third independent author (KK, GZ). Full texts were retrieved for those records considered eligible and for those where there was insufficient information from the abstract. For each included study data were extracted and reviewed by two of four independent authors using a pre-designed data extraction form (BA, JS, KK, RA). The extracted information included: title, authors, year of data collection, study design, study setting (inpatient, outpatient, and community-based), sample size, geographical region based in the World Bank region classification (25), World Bank country income classification [Low and middle income countries (LMIC) or High income countries (HIC)] (25), antipsychotic medication use (antipsychotic-naive cohort versus antipsychotic exposed and mixed SMI population), SMI diagnosis criteria, BMI classification criteria and prevalence estimates of overweight and obesity. When available in the studies we also extracted the prevalence of overweight and obesity by sex and for matched controls from the general population independently. Where there was more than one paper for the same study we selected the paper with the largest sample size or most recent date of publication (if the sample size was the same). In papers where the estimates of obesity were reported independently by country or type of SMI, they were separated and considered as independent studies.

### Risk of Bias

The quality of included studies was evaluated by two independent co-authors (KI, SR) according to criteria from the Joanna Briggs Institute (*JBI) Critical Appraisal Checklist* (26). An overall risk of bias assessment was conducted for each study following the procedure suggested by the Cochrane RoB tool (27). If all domains of the tool were scored as “yes” or only one of the domains was assessed as “unclear” the study was classified as “low risk of bias”; if two or three domains were assessed as “unclear” the study was classified as “medium risk of bias”; and if four or more were assessed as “unclear” or at least one was assessed as “no” the study was classified as “high risk of bias” (28).

### Data Analysis

#### Descriptive Analysis

Data from eligible studies were summarised providing information on the number and frequency of studies according to the World Bank income classification, the World Bank geographical region, type of SMI (schizophrenia, bipolar disorders, or a combination of SMI operationalized as “any”), the diagnostic tool to define the SMI, the study design, year of data collection, BMI classification, sex and overall risk of bias. Using standard procedures, we imputed the year of data collection, whenever this information was not available (29).

#### Meta-Analysis

We conducted three separate meta-analyses using random effect models to account for the high heterogeneity expected between the studies. The first determined the pooled prevalence of overweight, prevalence of obesity and combined prevalence of overweight and obesity. The second calculated the odds of overweight or obesity in people with SMI compared with the general population. The third estimated the odds of women with SMI having overweight and obesity compared to men with SMI. For the second and third meta-analysis, we used only the studies with available data on overweight and obesity for matched controls from the general population or men and women independently. Results are presented as odds ratios with 95% CI. We generated forest plots calculating the pooled prevalence and pooled odds ratios according to the type of SMI, setting, medication use, World Bank region, World Bank country income classification, and date of data collection.

Heterogeneity of studies was determined by inspecting forest plots and using the chi-square test for heterogeneity with a 10% level of statistical significance, and the I^2^ statistic. All analyses were performed using the metafor package in R V4.1.1 (Vienna, Austria) (30). We assessed publication bias by a visual inspection of a funnel plot of the log transformed proportion versus the standard error.

#### Sensitivity Analyses

To evaluate the robustness of the results we performed a series of sensitivity analyses. In the first analysis, we removed the studies assessed as “high risk of bias”. In the second, we removed studies that had an obesity cut-off point that differed from the WHO classification. In the third, we excluded studies with a sample size smaller than 323 (because this is the sample size needed to estimate the prevalence of obesity with a precision of 0.05 and confidence of 0.95, assuming a prevalence of 30%). We conducted the same set of analyses and compared the pooled estimate of obesity and heterogeneity with the data which included all studies (31).

We mapped the pooled prevalence of obesity by country in a world map. For countries with more than one study we calculated a weighted mean using data from all available studies. The maps were generated using the Rworldmap library in R V4.1.1 (Vienna, Austria) (30).

## Results

**Figure 1:**
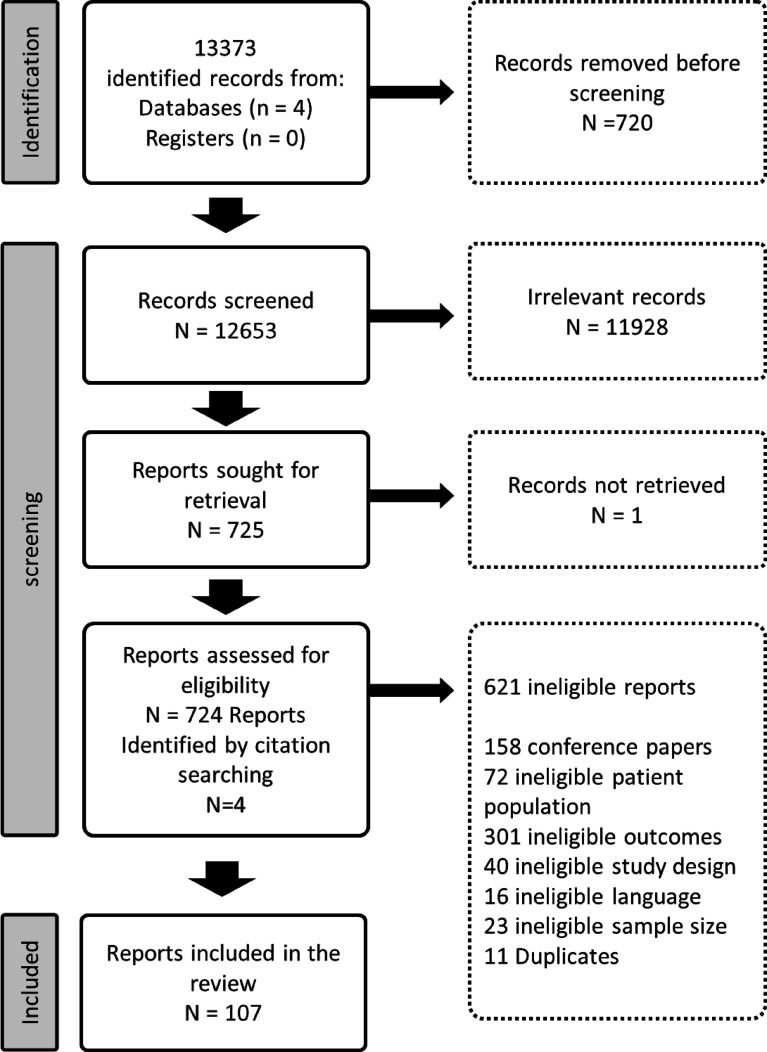
PRISMA chart.

As shown in the PRISMA chart (**Figure 1**) we screened 12,653 reports, identified 725 that fulfilled the inclusion criteria [one could not be retrieved (32)], and extracted data from 107 reports. Eight reports had duplicated data from the same cohort of people with SMI, we only kept the four reports with the most recent date of publication. We separated the prevalence of obesity for each country from a report that provided obesity estimates in 10 European countries and one from six Asian countries. We also separated the prevalence of obesity for each SMI condition in three reports that provided estimates separately for people with schizophrenia and bipolar disorders. We extracted 120 estimates of obesity, 108 estimates of overweight, 24 estimates of the prevalence of obesity in the general population and 30 estimates of obesity for men and women separately. The complete list of the papers is provided in the **Supplementary Material**. We found less than 10% of disagreements in the title and abstract and full text screening and less than 5% in data extraction, indicating good interrater reliability.

**Table 1** summarises characteristics of the 107 papers providing 120 estimates of obesity (called studies hereafter), including 139,282 people with SMI. The sample size of the studies ranged from 30 to 43,093 with a mean of 267. The sample ages ranged from 21.3 years to 70.4 years with a mean of 41.3 years. We found 95 studies (79.1%) from HICs and 25 (20.1%) from LMICs, including data from 43 countries; more than 50% of the studies were conducted in the regions of Europe and North America, whilst the percentage of studies from the regions of Sub-Saharan Africa (2.4%), South Asia (4.0%), Middle East and North Africa (1.6%), and Latin America and the Caribbean (4.8%) were low. More than 80% of the studies classified SMI according to DSM and ICD criteria and 90% of the studies used the WHO classification of overweight and obesity.

**Table 1:** Summary of the studies.

Variable	N (120)	Percentage
**World Bank classification**		
HICs	95	79.1%
LMICs	25	20.1%
**World Bank region**		
Sub Saharan Africa	3	2.4%
East Asia and Pacific	32	25.8%
North America	31	25%
Europe and Central Asia	45	36.3%
South Asia	5	4.0%
Middle East and North Africa	2	1.6%
Latin America and Caribbean	6	4.8%
**Type of SMI**		
Any SMI**^1^**	27	22.5%
Schizophrenia**^2^**	71	59.1%
Bipolar Disorder	22	18.3%
**Setting**		
Inpatient	33	26.6%
Outpatient	61	49.2%
In- and Outpatient	24	19.4%
Community	6	4.8%
**Antipsychotic medication**		
Prescribed antipsychotic medication^3^	111	89.5%
Antipsychotic-naive population	7	5.6%
Not reported	6	4.8%
**SMI diagnostic tool**		
DSM	78	65.0%
ICD	18	15.0%
DSM and ICD	3	2.5%
Not specified	21	17.5%
**Study design**		
Cross-sectional	110	91.6%
Cohort	6	5.0%
Prospective longitudinal	4	3.3%
**Year of data collection**		
1990 - 1999	1	0.8%
2000 - 2009	39	32.5%
2010 - Date	80	66.6%
**BMI classification**		
World Health Organization	109	90.8%
Taiwan standards	3	2.5%
Japanese Society for the Study of Obesity	3	2.5%
Indonesian Standards	1	0.8%
Working Group on Obesity China	1	0.8%
Asia Standard Scale	3	2.4%
**Overall risk of bias**		
Low	70	58.3%
Medium	27	22.5%
High	23	19.1%

HIC, High income country; LMIC, Low- and middle-income countries; SMI, Severe mental illness. ^1^The “any” category includes studies where the estimates of obesity were not separated between the type SMI; ^2^we did not find studies focusing on major depression with psychotic features independently and only found one study focusing at first episode of psychosis which was included in the “schizophrenia” category; ^3^Studies including participants with prescribed antipsychotic medication or open population (mix of participants taking and not taking antipsychotic medication).

### Risk of Bias

Fifty eight percent of the studies were classified as having “low” risk of bias, 22.5% as “medium” risk of bias and almost 20% were classified as having “high” risk of bias (**Table 1** and **Supplementary Material**).

### Prevalence of Overweight and Obesity in People With SMI

**Figure 2:**
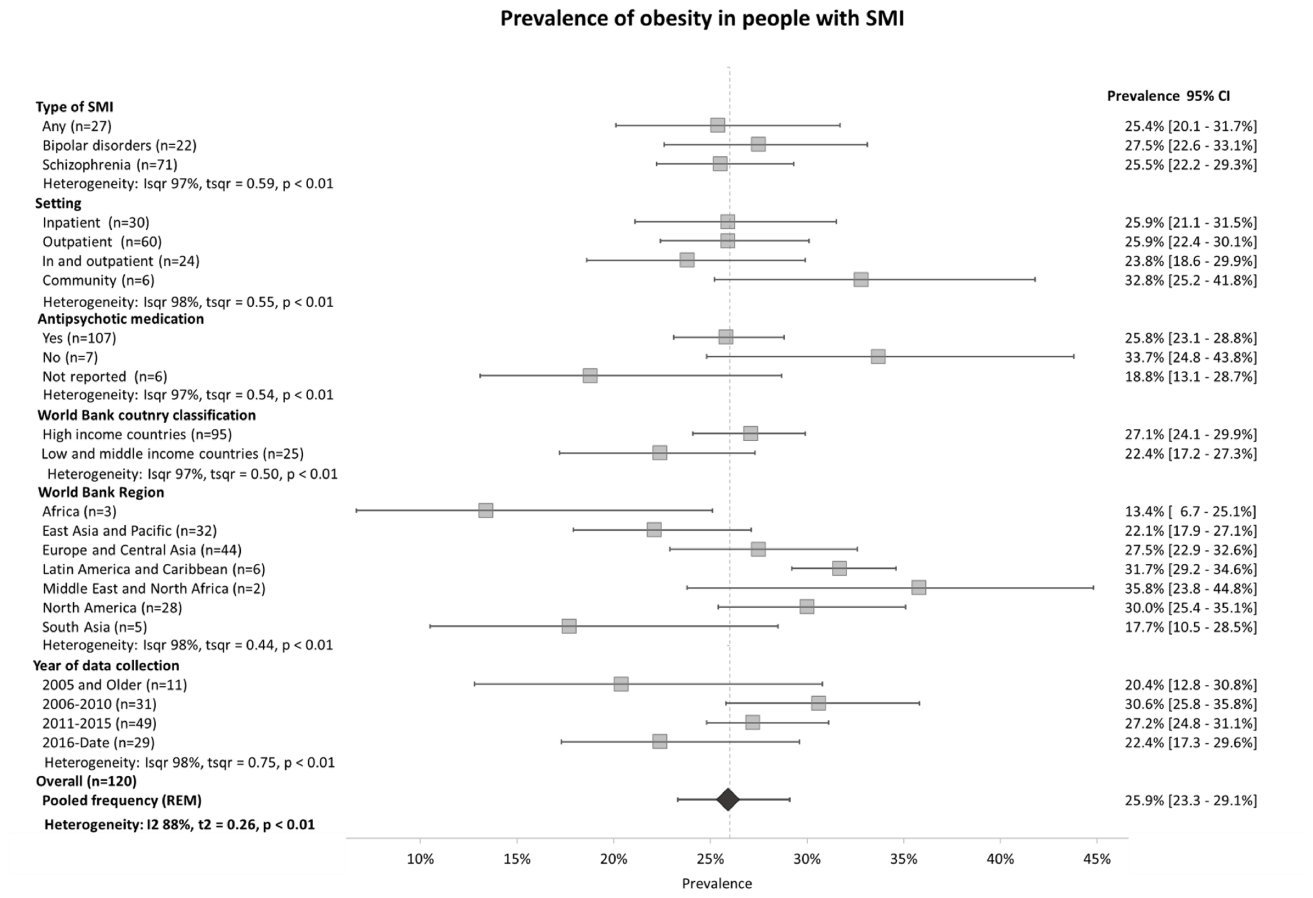
Prevalence of obesity in people with SMI according to type of SMI, region, World Bank classification and year of data collection.

As shown in **Figure 2** and **Table 2**, the pooled prevalence of obesity in this population was 25.9% (95% C.I. = 23.3-29.1) and the combined prevalence of overweight and obesity was 60.1% (95% C.I. = 55.8-63.1). The pooled prevalence of obesity was 22.4% (95% C.I. = 17.2-27.3) in LMICs and 27.1% (95% C.I. = 24.1-29.9) in HICs, and the combined prevalence of overweight and obesity was 55.7% (95% C.I. = 49.5-61.7) in LMICs and 60.7% (95% C.I. = 56.9-64.2) in HICs. The region with the lowest prevalence of obesity was Sub-Saharan Africa with a pooled prevalence of 13.3% (95% C.I. = 6.7-25.1), followed by South Asia with a pooled prevalence of 17.7% (95% C.I. = 10.5-28.5). The regions with the highest prevalence of obesity were the Middle East and North Africa with obesity prevalence of 35.8% (95% C.I. = 23.8-44.8) and a combined overweight and obesity prevalence of 71.4% (95% C.I. = 67.8-74.7). There were no differences in the prevalence of obesity between studies performed in antipsychotic-naive populations [33.7% (95% C.I. = 24.8-43.8)] and those in people prescribed antipsychotics [30.6% (95% C.I. = 29.4-32.1)]. There were no substantial differences in the prevalence of overweight and obesity between the types of SMI and between inpatient, outpatient, or community settings. We found substantial heterogeneity (I^2^ = 88-98%, p<0.01) in overall estimates, which was not reduced in the subgroup estimates (I^2^ = 97-98%, p<0.01).

**Table 2:** Pooled prevalence of overweight obesity and combined overweight and obesity according to SMI, geographical region, World Bank classification and year of publication.

	Overweight	Obesity	Combined prevalence of overweight and obesity
Number of studies	108	120	108
Overall pooled prevalence (95%CI)	31.2% (28.9-32.1)	25.9% (23.3-29.1)	60.1% (55.8-63.1)
Heterogeneity I^2^	97.1%	88.3%	98.1%
*T* ^2^	0.11	0.55	0.47
P	<0.01	<0.01	<0.01
**Type of SMI**			
Any SMI**^1^**	32.9% (31.2-34.7)	25.4% (20.1-31.7)	61.2% (56.3-65.7)
Bipolar disorder	31.1% (28.8-33.8)	27.5% (22.6-33.1)	60.7% (55.8-65.2)
Schizophrenia	29.8% (27.4-32.2)	25.5% (22.2-29.3)	58.6% (53.5-63.6)
**Setting**			
Inpatient	31.2% (28.2-33.9)	25.9% (21.1-31.5)	60.1% (54.4-67.2)
Outpatient	30.1% (28.4-33.1)	25.9% (22.4-30.1)	58.8% (53.8-65.1)
In- and outpatient	31.4% (29.1-34.3)	23.8% (18.6-29.9)	58.5% (51.8-65.1)
Community	29.8% (26.6-33.9)	32.8% (25.2-41.8)	63.2% (55.3-70.5)
**Antipsychotic medication**			
Prescribed antipsychotic medication^2^	30.6% (29.4-32.1)	25.8% (23.1-28.8)	59.3% (55.8-62.6)
Antipsychotic-naive population	34.7% (32.2-37.4)	33.7% (24.8-43.8)	68.2% (59.7-75.7)
Not reported	30.9% (19.2-45.7)	18.8% (13.1-28.7)	53.7% (37.7-69.0)
**World Bank Region**			
East Asia and Pacific	28.4% (25.4-31.6)	22.5% (17.9-27.1)	53.8% (46.8-60.9)
East Asia and Pacific (WHO)^3^	28.2% (24.7-32.3)	18.1% (14.6-24.1)	51.2% (44.3-59.3)
Europe and Central Asia	31.9% (28.9-35.1)	27.5% (22.9-32.6)	63.1% (56.3-69.7)
Latin America & Caribbean	34.5% (31.6-37.5)	31.7% (29.2-34.6)	65.2% (60.0-70.1)
Middle East and North Africa	34.2% (25.5-44.2)	35.8% (23.8-44.8)	71.4% (67.8-74.7)
North America	31.1% (29.1-33.9)	30% (25.4-35.1)	62.8% (58.2-66.9)
South Asia	31.1% (21.7-41.3)	17.7% (10.5-28.5)	51.3% (40.9-61.6)
Sub-Saharan Africa	33.1% (27.8-38.7)	13.4% (6.7-25.1)	47.9% (43.7-52.3)
**World Bank Classification**			
HICs	30.0% (29.1-32.1)	27.1% (24.1-29.9)	60.7% (56.9-64.2)
LMICs	32.1% (29.7-35.2)	22.4% (17.2-27.3)	55.7% (49.5-61.7)
**Year of data collection**			
2005 and Older	27.2% (22.9-31.9)	20.4% (12.8-30.8)	51.1% (38.3-63.8)
2006 - 2010	33.2% (30.0-36.6)	30.6% (25.8-35.8)	66.0% (60.5-71.1)
2011 - 2015	30.6% (28.2-33.2)	27.2% (24.8-31.1)	59.5% (54.6-64.1)
2016 - Date	29.5% (27.2-32.8)	22.4% (17.2-29.6)	55.3% (48.9-61.5)

^1^The “any” category includes studies where the estimates of obesity were not separated between the type SMI, we did not find studies looking at major depression with psychotic features independently and only found one study looking at first episode of psychosis which was included in the “schizophrenia” category; ^2^Studies including participants with prescribed antipsychotic medication or open population (mix of participants taking and not taking antipsychotic medication); ^3^Prevalence from the sensitivity analysis excluding papers with cut-of scores different than the WHO.

**Figure 3:**
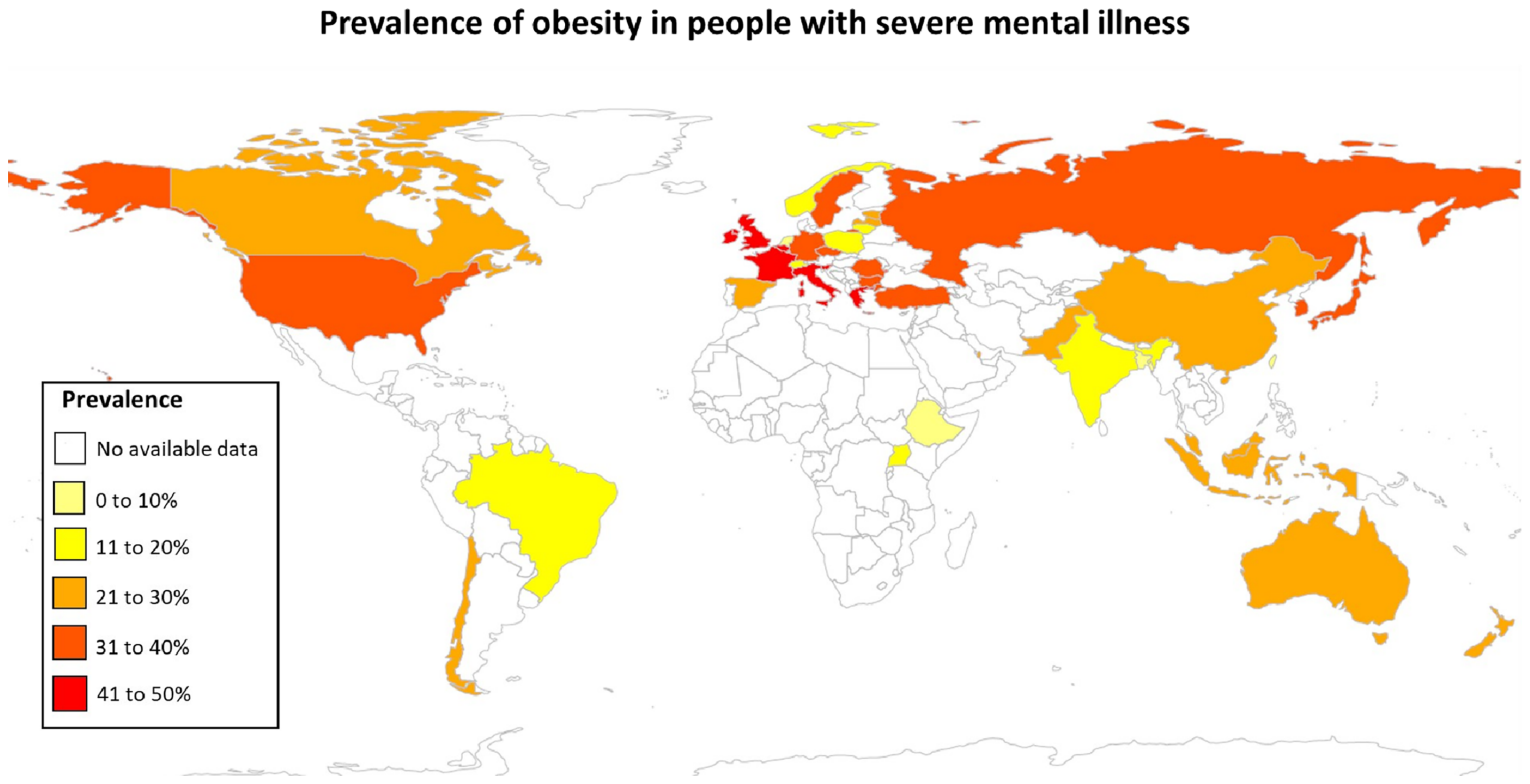
Geographical variation in the prevalence of obesity in people with SMI.

As shown in **Figure 3**, the countries with the highest prevalence of obesity (over 40%) were Germany, Italy and the UK. While the countries with the lowest reported prevalence were Ethiopia and Uganda. There were no available estimates in most of the countries in the regions of Africa, the Middle East and Latin America.

**Figure 4:**
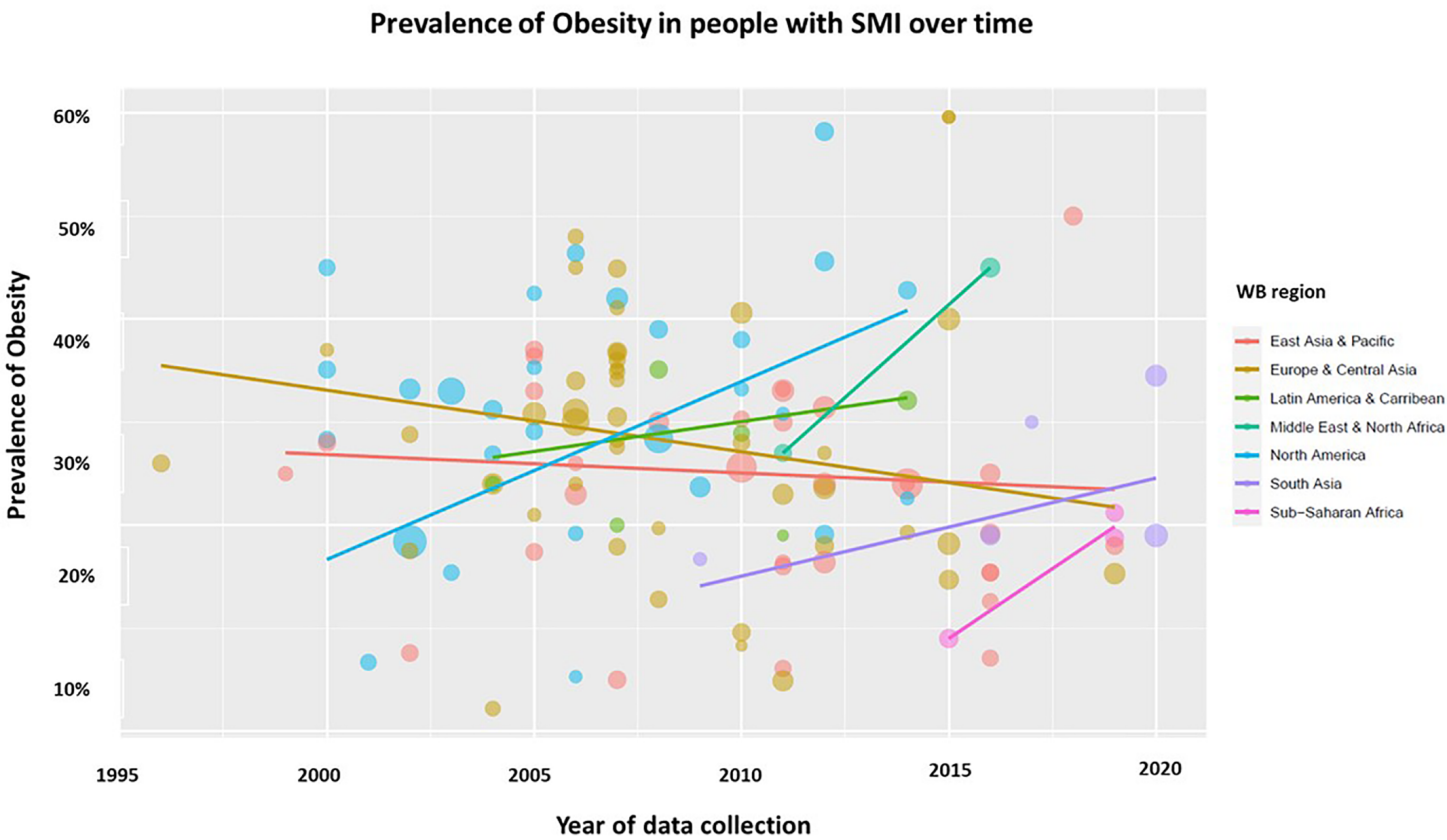
Weighted linear regression models between the prevalence of obesity and time according to World Bank region. Data on global trends in the prevalence of obesity in people with SMI from 1995 to 2020 from the World Bank regions of the world show differences in the trend of the prevalence of obesity. The area of the dots corresponds to the sample size of each study. WB, World Bank; SMI, Severe mental illness.

**Figure 4** shows trend lines of the prevalence of obesity in each geographical World Bank region over time. There was a decreasing trend in the prevalence of obesity in the region of Europe and Central Asia; in contrast there was an increasing trend in the regions of Latin America and the Caribbean, Middle East and North Africa, North America, South Asia and Sub-Saharan Africa.

### Obesity in People With SMI Compared With the General Population

**Figure 5:**
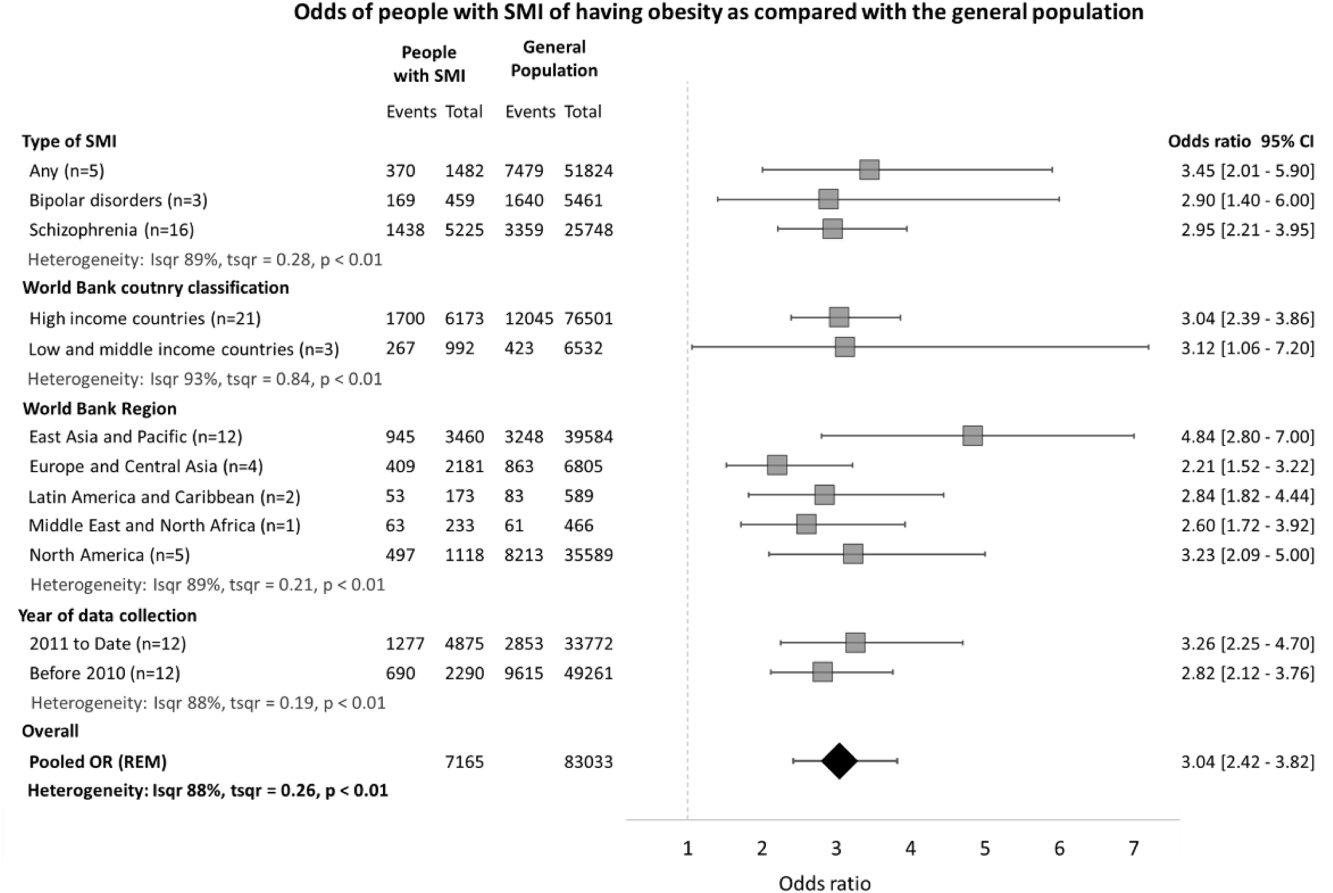
Odds of people with SMI of having obesity as compared to the general population.

Twenty four studies reported the prevalence of obesity in the general population by using matched controls. As seen in **Table 3**, people with SMI were 3.04 (95% C.I. = 2.42 - 3.82) times more likely to have obesity and 2.03 (95% C.I. = 1.60 - 2.59) times more likely to have either overweight or obesity compared with the general population. People with SMI in the East Asia and Pacific region were 4.84 (95% C.I. = 2.80 - 7.00) times more likely to have obesity than people in the general population, while people with SMI from Europe and Central Asia had 2.21 (95% C.I. = 1.52 - 3.22) higher odds (**Figure 5**). There were no differences in the odds of people with SMI being overweight compared with the general population. We found high heterogeneity between the studies both in the overall (88%) and subgroup estimates (88-93%).

**Table 3:** Odds of people with SMI of having overweight and obesity as compared with the general population.

	Number of studies	N people with SMI	N General population	Odds ratio	95% CI^1^	p	I^2 2^	*T* ^2 3^
Overweight	21	6478	78937	1.07	0.91-1.27	<0.01	81%	0.11
Obesity	24	7165	83033	3.04	2.42 - 3.82	<0.01	91%	0.42
Overweight and obesity	21	6478	78937	2.03	1.60-2.59	<0.01	92%	0.27

^1^95% confidence interval; ^2^I squared test for heterogeneity; ^3^Tau squared test for heterogeneity.

### Sex Differences in the Prevalence of Obesity in People With SMI

**Figure 6:**
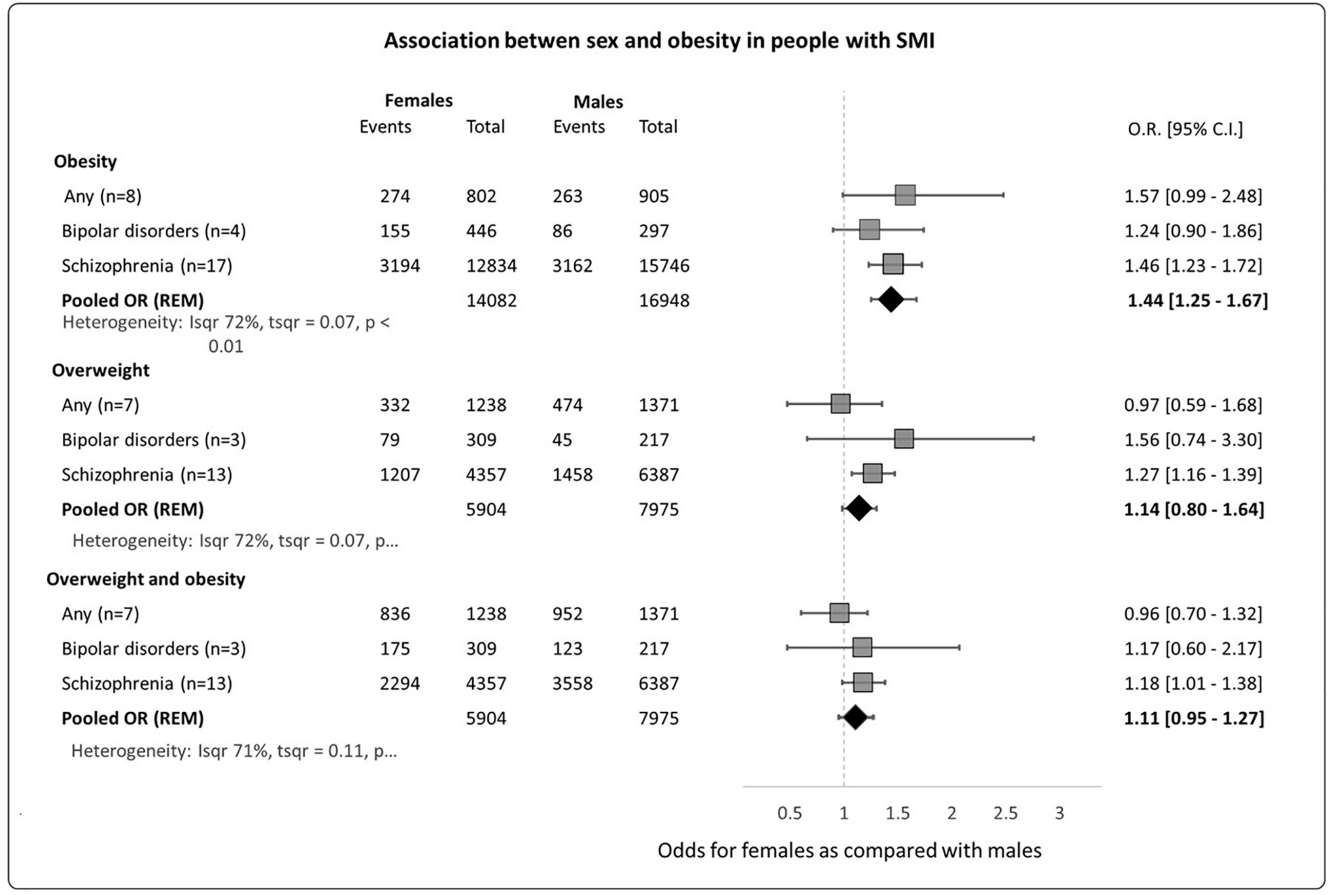
Association between sex and obesity in people with SMI.

As shown in **Figure 6**, twenty four studies reported obesity outcomes independently for men and women; the meta-analysis from these studies showed that women with schizophrenia were 1.46 (95% C.I. = 1.23-1.72) times more likely to have obesity and 1.27 (95% C.I. = 1.16-1.39) more likely to be overweight compared with men with schizophrenia. There were no differences in the odds of obesity between men and women with bipolar disorder or any SMI.

### Sensitivity Analysis and Publication Bias

The pooled prevalence of obesity did not change substantially (more than 1.2%) when studies with high risk of bias, BMI classification other than WHO, and small sample sizes were removed. The model excluding studies with high risk of bias (n= 95) had a pooled estimate of obesity of 25.6% (95% C.I. = 22.8-28.7%;I^2^ = 98.2, t^2^ = 0.57); the model excluding studies (n=109) with cut-off values different from the WHO gave a pooled prevalence of 25.3% (22.7 - 28.2%; I^2^ = 97.9, t^2^ = 0.56); and the model excluding studies with small sample sizes (n=43) showed a pooled prevalence of 27.1% (22.8 = 38.1%; I^2^ = 99.1, t^2^ = 0.58). As shown in the funnel plot (see **Supplementary Material**), there was no evidence of publication bias.

## Discussion

### Main Findings

This review provides the first comprehensive overview of the prevalence of overweight and obesity among people with SMI, with data from 43 countries from all regions of the world. We found a high prevalence of overweight and obesity and higher odds of people with SMI living with obesity than the general population in all regions. There were differences in the prevalence of obesity according to the geographical region, country income classification and sex. We also found regional differences in the trend of obesity in this population. There was a disproportionately low number of studies from LMICs, particularly from Sub-Saharan Africa, the Middle East and Latin American regions.

### Prevalence of Overweight and Obesity

The high prevalence of overweight and obesity found in this SMI population and the higher odds of obesity compared with the general population is consistent with epidemiological data, where the prevalence of obesity in the general population was lower than the prevalence found in people with SMI across all regions of the world such as Africa (12.7%), Americas (27%), Europe (22.5%), and West Pacific and East asia (5.4%) (33). The high prevalence of obesity found in people with SMI is likely to be linked to the use of antipsychotic medications and unhealthy lifestyle behaviours which are more common in SMI (34–36). Despite the high prevalence of obesity and the excess mortality, studies have consistently shown that this population has poorer access to health risk modification advice and lifestyle interventions to prevent and treat obesity compared with the general population (37). These disparities are even greater in LMICs, where poverty affects a higher proportion of the SMI population and where access to healthcare is even more limited, and where evidence of availability and effectiveness of lifestyle intervention among people with SMI is nearly non-existent (38, 39). Implementation of screening programmes assessing overweight and obesity might be useful to identify people at higher risk, and referral to lifestyle interventions and collaborative care as suggested by the WHO guidelines for management of physical conditions in adults with SMI (8). Despite the multiple barriers faced by this population such as stigma, illness symptoms and low motivation, the few pharmacological and lifestyle interventions available to prevent and treat obesity in this population have proven to be effective to maintain and reduce body weight (39–42). In addition, they have shown benefits in other parameters such as fasting blood glucose, hypertension and dyslipidemia (43). Thus, lifestyle and pharmacological interventions could provide a feasible and cost-effective solution to reduce obesity, prevent non-communicable diseases (NCDs) and contribute to reduction of the mortality gap seen in this population (44).

The geographical differences in the prevalence and trends of obesity are also found in the general population (33), and may be associated with socio-economic inequalities, lifestyle, food availability and other environmental and genetic factors in each region (45). These differences should be taken into consideration in developing tailored interventions and programmes to prevent and treat obesity in each country or region (46). Special attention should be placed in countries and regions where the prevalence of obesity is high and where there is an increasing trend in the prevalence. An unexpected finding of the review was the higher prevalence of obesity (although not statistically significant) in studies conducted in an antipsychotic naive population compared with studies where the participants were on psychotropic medication. These findings should be interpreted with caution since the estimate of obesity for antipsychotic naive populations comes from only seven studies conducted in Europe and North America, which reported a high prevalence of obesity.

The sex differences concerning the burden of overweight and obesity are also seen in the general population (47), where the prevalence of overweight and obesity is higher among women especially in LMICs (36, 48). Studies looking specifically into gender differences have found fewer gender differences in health outcomes in people with bipolar disorder (49) than those with schizophrenia (50, 51). It has been suggested that interventions to prevent and treat obesity should be tailored to the sex of the participant to increase their acceptability and effectiveness (52).

### Disparities Between HICs and LMICs

Even if we consider the papers other than English (**Supplementary Material**), we found a disproportionately low number of studies from LMICs and, in particular, a big gap in the evidence available from Sub-Saharan Africa and Latin American regions. These disparities in the contribution of LMICs in health research have been highlighted in other health disciplines (53, 54). Possible explanations could include challenges such as stigmatized health conditions, inadequate health care infrastructure, lack of national budget designated to the mental health sector and research budget (55). Collecting information on the prevalence of obesity and other physical conditions is needed in these regions to develop health policy and tailored programmes and interventions that might improve health and reduce inequalities in this population. Approaches such as the “Investigating Mental and Physical Health Together” (IMPACT) SMI survey could be used to collect and analyse data on obesity, NCDs and health risk behaviours in this population in a cost-effective manner in a budget restricted setting (56).

### Strengths and Limitations

There are some limitations that require acknowledgment. First, because of the diversity in the regions, type of SMI and setting, there was substantial heterogeneity between the studies, which could not be reduced by stratifying the sample. However, we did not find evidence of publication bias and performed sensitivity analyses that supported the robustness of our results. Second, there was not a consistent definition of obesity across studies conducted in Asia which may have led to the overestimation of the prevalence of obesity, to address this we conducted sensitivity analysis excluding these studies and providing the data of the prevalence of obesity using only these studies. Third, there were also minor deviations from the protocol, such as the inclusion of papers written in all languages, which was not possible due to resources and time constraints, this may have affected the estimates of obesity especially in China, Turkey and Russia (were most of these publications in other languages came from – A list of these publications are available in the **Supplementary Material**-); the use of any validated definition of obesity rather than only the WHO, which was changed to be more inclusive particularly studies conducted in Asia; we stated we would only conduct a meta-analysis if the heterogeneity between the studies was low, however after inspection of the data we decided to provide an estimate acknowledging this limitation. Fourth, we could not account for unmeasured confounders such as diet and physical activity that could have affected the prevalence of obesity in each study. Despite these limitations this is the first study to examine the prevalence of obesity and overweight in people with SMI from a global perspective estimating the prevalence and trend of obesity in every region of the world.

## Conclusion

People with SMI have a high prevalence of obesity and higher odds of having obesity than the general population. This may be contributing to the higher morbidity and mortality seen in this population. This study demonstrated regional and sex differences in the prevalence and trends of obesity. The progression from obesity to metabolic diseases and premature mortality in this population should be assessed in more detail. People with SMI around the world could benefit from screening programs and tailored interventions to reduce and prevent obesity.

## Data Availability Statement

The raw data supporting the conclusions of this article will be made available by the authors, without undue reservation.

## Author Contributions

GZ, MA, NS, and RH designed the study. GZ and MA drafted the manuscript. MB, NS and RH made substantial contributions to conception of the manuscript and interpretation of data. AA, BA, FA, HK, JS, KI, KK, NT, RA, and SR made substantial contributions to screening and data extraction and BA, GZ, and RA conducted the analysis. MB, NS, and RH critically revised the manuscript. All authors contributed to the article and approved the submitted version.

## Funding

This research was funded by the National Institute for Health Research (NIHR) (Grant: GHRG 17/63/130): using UK aid from the UK Government to support global health research. The views expressed in this publication are those of the author(s) and not necessarily those of the NIHR or the UK Department of Health and Social Care.

## Conflict of Interest

The authors declare that the research was conducted in the absence of any commercial or financial relationships that could be construed as a potential conflict of interest.

## Publisher’s Note

All claims expressed in this article are solely those of the authors and do not necessarily represent those of their affiliated organizations, or those of the publisher, the editors and the reviewers. Any product that may be evaluated in this article, or claim that may be made by its manufacturer, is not guaranteed or endorsed by the publisher.
