# Supplementary material for: Prevalence of Overweight and Obesity in People With Severe Mental Illness: Systematic Review and Meta-Analysis
Source: Front Endocrinol (Lausanne). 2021 Nov 25;12:769309. doi: 10.3389/fendo.2021.769309 (PMC8656226; doi:10.3389/fendo.2021.769309)
Supplement: Supplementary file 1 [file DataSheet_1.zip › Supplementary Material.docx]

**This is an electronic supplementary material accompanying the paper “Prevalence of overweight and obesity in people with severe mental illness: systematic review and meta analysis”**

**Supplementary Table 1.** Characteristics of the eligible studies

| **Author** | **Sample size** | **SMI^a^** | **Setting** | **Country** | **WB region** | **WB income classification** | **Study design** | **Comparison with the general population** | **Comparison Males and Females** | **Overall risk of bias** |
| --- | --- | --- | --- | --- | --- | --- | --- | --- | --- | --- |
| Silversone 1988 [(1)](https://paperpile.com/c/3JPyg8/WFk4) | 226 | Schizophrenia | Outpatient | England | Europe & Central Asia | HIC | Cross-sectional | No | Yes | Medium |
| Elmslie 2000 [(2)](https://paperpile.com/c/3JPyg8/IbKa) | 89 | Bipolar disorders | Out and Inpatient | New Zealand | East Asia & Pacific | HIC | Cross-sectional | Yes | Yes | Low |
| Coodin 2001 [(3)](https://paperpile.com/c/3JPyg8/ax5V) | 183 | Schizophrenia | Inpateint | Canada | North America | HIC | Cross-sectional | Yes | No | High |
| Davidson 2001 [(4)](https://paperpile.com/c/3JPyg8/m6UI) | 234 | Any | Outpatient | Australia | East Asia & Pacific | HIC | Cross-sectional | Yes | Yes | High |
| Winkelman 2001 [(5)](https://paperpile.com/c/3JPyg8/W5VZ) | 46 | Schizophrenia | Outpatient | US | North America | HIC | Cross-sectional | No | No | Medium |
| Winkelman 2001 [(5)](https://paperpile.com/c/3JPyg8/W5VZ) | 92 | Bipolar disorders | Outpatient | US | North America | HIC | Cross-sectional | No | No | Medium |
| Strassnig 2003 [(6)](https://paperpile.com/c/3JPyg8/LxbK) | 146 | Schizophrenia | Outpatient | US | North America | HIC | Cross-sectional | No | No | Low |
| Daumit 2003 [(7)](https://paperpile.com/c/3JPyg8/aqlu) | 223 | Any | Inpateint | US | North America | HIC | Cross-sectional | Yes | Yes | High |
| Hsiao 2004 [(8)](https://paperpile.com/c/3JPyg8/GLSP) | 201 | Schizophrenia | Outpatient | Taiwan | East Asia & Pacific | LMIC | Cross-sectional | Yes | Yes | High |
| Strassnig 2005 [(9)](https://paperpile.com/c/3JPyg8/x7wX) | 143 | Schizophrenia | Outpatient | US | North America | HIC | Cross-sectional | No | No | Low |
| Fagiolini 2005 [(10)](https://paperpile.com/c/3JPyg8/jlh6) | 171 | Bipolar disorders | Outpatient | US | North America | HIC | Cross-sectional | No | No | Low |
| Wang 2006 [(11)](https://paperpile.com/c/3JPyg8/cvvW) | 377 | Bipolar disorders | Community | US | North America | HIC | Cross-sectional | No | Yes | High |
| DeHert 2006 [(12)](https://paperpile.com/c/3JPyg8/wBLd) | 300 | Any | Out and Inpatient | Belgium | Europe & Central Asia | HIC | Cross-sectional | No | No | High |
| Dickerson 2006 [(13)](https://paperpile.com/c/3JPyg8/xla1) | 169 | Any | Outpatient | US | North America | HIC | Cross-sectional | Yes | Yes | Low |
| Faulkner 2007 [(14)](https://paperpile.com/c/3JPyg8/pS4H) | 90 | Schizophrenia | Inpateint | Canada | North America | HIC | Cross-sectional | No | No | Low |
| Henderson 2006 [(15)](https://paperpile.com/c/3JPyg8/WLQW) | 88 | Schizophrenia | Out and Inpatient | US | North America | HIC | Cross-sectional | No | No | High |
| Kitabayashi 2006 [(16)](https://paperpile.com/c/3JPyg8/KLPb) | 273 | Schizophrenia | Inpateint | Japan | East Asia & Pacific | HIC | Cross-sectional | Yes | Yes | Low |
| Leitao-azevedo 2006 [(17)](https://paperpile.com/c/3JPyg8/Uh1h) | 121 | Schizophrenia | Outpatient | Brazil | Latin America & Carribean | HIC | Cross-sectional | No | No | Low |
| Feeney 2007 [(18)](https://paperpile.com/c/3JPyg8/sVIc) | 122 | Schizophrenia | Out and Inpatient | Ireland | Europe & Central Asia | HIC | Cross-sectional | No | No | Medium |
| Hellerstein 2007 [(19)](https://paperpile.com/c/3JPyg8/UdZY) | 69 | Schizophrenia | Outpatient | England | Europe & Central Asia | HIC | Cross-sectional | No | Yes | Medium |
| Kim 2008 [(20)](https://paperpile.com/c/3JPyg8/CNUI) | 179 | Bipolar disorders | Inpateint | South Korea | East Asia & Pacific | HIC | Cross-sectional | No | No | Medium |
| Mackin 2007 [(21)](https://paperpile.com/c/3JPyg8/5A5h) | 106 | Any | Outpatient | England | Europe & Central Asia | HIC | Cross-sectional | No | Yes | Low |
| Nishiyama 2007 [(22)](https://paperpile.com/c/3JPyg8/vgrR) | 208 | Schizophrenia | Inpateint | Japan | East Asia & Pacific | HIC | Cross-sectional | Yes | Yes | Medium |
| Tirupati 2007 [(23)](https://paperpile.com/c/3JPyg8/NRi2) | 221 | Any | Out and Inpatient | Australia | East Asia & Pacific | HIC | Cross-sectional | No | No | High |
| Maina 2008 [(24)](https://paperpile.com/c/3JPyg8/ndYc) | 76 | Bipolar disorders | Out and Inpatient | Italy | Europe & Central Asia | HIC | Cross-sectional | No | No | High |
| Ainsah 2008 [(25)](https://paperpile.com/c/3JPyg8/yWpy) | 97 | Schizophrenia | Outpatient | Malaysia | East Asia & Pacific | LMIC | Cross-sectional | No | No | High |
| Correll 2008 [(26)](https://paperpile.com/c/3JPyg8/Etl1) | 185 | Any | Inpateint | US | North America | HIC | Cross-sectional | No | No | High |
| Limosin 2008 [(27)](https://paperpile.com/c/3JPyg8/2FIv) | 5962 | Schizophrenia | Out and Inpatient | France | Europe & Central Asia | HIC | Cross-sectional | No | Yes | Low |
| McLaren 2008 [(28)](https://paperpile.com/c/3JPyg8/bdBM) | 5137 | Any | Out and Inpatient | Canada | North America | HIC | Cross-sectional | No | No | Medium |
| VanWinkel 2008 [(29)](https://paperpile.com/c/3JPyg8/CXkz) | 60 | Bipolar disorders | Outpatient | Belgium | Europe & Central Asia | HIC | Cross-sectional | No | No | High |
| Calkin 2009 [(30)](https://paperpile.com/c/3JPyg8/2WTk) | 276 | Bipolar disorders | Inpateint | Canada | North America | HIC | Cross-sectional | No | No | Medium |
| Bell 2009 [(31)](https://paperpile.com/c/3JPyg8/cDmN) | 819 | Schizophrenia | Outpatient | US | North America | HIC | Cross-sectional | No | No | Low |
| Bernado 2009 [(32)](https://paperpile.com/c/3JPyg8/9VVm) | 733 | Schizophrenia | Inpateint | Spain | Europe & Central Asia | HIC | Cross-sectional | No | No | Low |
| Boden 2009 [(33)](https://paperpile.com/c/3JPyg8/0Njp) | 59 | Schizophrenia | Outpatient | Sweden | Europe & Central Asia | HIC | Cohort | No | No | Low |
| deAlmeida 2009 [(34)](https://paperpile.com/c/3JPyg8/xV1z) | 84 | Bipolar disorders | Outpatient | Brazil | Latin America & Carribean | LMIC | Cross-sectional | No | No | Medium |
| Fiedorowicz 2008 [(35)](https://paperpile.com/c/3JPyg8/TQkj) | 217 | Bipolar disorders | Inpateint | US | North America | HIC | Cross-sectional | Yes | No | High |
| Huang 2009 [(36)](https://paperpile.com/c/3JPyg8/NJk8) | 650 | Schizophrenia | Inpateint | Taiwan | East Asia & Pacific | LMIC | Cross-sectional | No | Yes | Low |
| UmaDevi 2009 [(37)](https://paperpile.com/c/3JPyg8/nXRp) | 60 | Schizophrenia | Inpateint | India | South Asia | LMIC | Cross-sectional | No | No | High |
| Vancampfort 2011 [(38)](https://paperpile.com/c/3JPyg8/E3I2) | 60 | Schizophrenia | Inpateint | Belgium | Europe & Central Asia | HIC | Cross-sectional | No | No | High |
| Correll 2010 [(39)](https://paperpile.com/c/3JPyg8/ErP7) | 10084 | Any | Outpatient | US | North America | HIC | Cross-sectional | No | No | Low |
| Lee 2010 [(40)](https://paperpile.com/c/3JPyg8/zl2u) | 152 | Bipolar disorders | Out and Inpatient | South Korea | East Asia & Pacific | HIC | Cross-sectional | Yes | Yes | Low |
| Mahendran 2010 [(41)](https://paperpile.com/c/3JPyg8/brfa) | 266 | Schizophrenia | Outpatient | Singapore | East Asia & Pacific | HIC | Cohort | No | Yes | High |
| Arango 2011 [(42)](https://paperpile.com/c/3JPyg8/rfEw) | 1452 | Schizophrenia | Outpatient | Spain | Europe & Central Asia | HIC | Cross-sectional | No | No | Medium |
| Chu 2011 [(43)](https://paperpile.com/c/3JPyg8/dCe9) | 878 | Schizophrenia | Inpateint | Taiwan | East Asia & Pacific | LMIC | Cross-sectional | No | No | Low |
| Falissard 2011 [(44)](https://paperpile.com/c/3JPyg8/QOgK) | 484 | Schizophrenia | Outpatient | Bulgaria | Europe & Central Asia | LMIC | Cross-sectional | No | No | Low |
| Falissard 2011 [(44)](https://paperpile.com/c/3JPyg8/QOgK) | 163 | Schizophrenia | Outpatient | Czech Republic | Europe & Central Asia | HIC | Cross-sectional | No | No | Low |
| Falissard 2011 [(44)](https://paperpile.com/c/3JPyg8/QOgK) | 106 | Schizophrenia | Outpatient | Estonia | Europe & Central Asia | HIC | Cross-sectional | No | No | Low |
| Falissard 2011 [(44)](https://paperpile.com/c/3JPyg8/QOgK) | 57 | Schizophrenia | Outpatient | Germany | Europe & Central Asia | HIC | Cross-sectional | No | No | Low |
| Falissard 2011 [(44)](https://paperpile.com/c/3JPyg8/QOgK) | 261 | Schizophrenia | Outpatient | Greece | Europe & Central Asia | HIC | Cross-sectional | No | No | Low |
| Falissard 2011 [(44)](https://paperpile.com/c/3JPyg8/QOgK) | 145 | Schizophrenia | Outpatient | Italy | Europe & Central Asia | HIC | Cross-sectional | No | No | Low |
| Falissard 2011 [(44)](https://paperpile.com/c/3JPyg8/QOgK) | 119 | Schizophrenia | Outpatient | Latvia | Europe & Central Asia | HIC | Cross-sectional | No | No | Low |
| Falissard 2011 [(44)](https://paperpile.com/c/3JPyg8/QOgK) | 195 | Schizophrenia | Outpatient | Lithuania | Europe & Central Asia | HIC | Cross-sectional | No | No | Low |
| Falissard 2011 [(44)](https://paperpile.com/c/3JPyg8/QOgK) | 92 | Schizophrenia | Outpatient | Romania | Europe & Central Asia | LMIC | Cross-sectional | No | No | Low |
| Falissard 2011 [(44)](https://paperpile.com/c/3JPyg8/QOgK) | 383 | Schizophrenia | Outpatient | Russia | Europe & Central Asia | HIC | Cross-sectional | No | No | Low |
| Falissard 2011 [(44)](https://paperpile.com/c/3JPyg8/QOgK) | 91 | Schizophrenia | Outpatient | Slovenia | Europe & Central Asia | HIC | Cross-sectional | No | No | Low |
| Falissard 2011 [(44)](https://paperpile.com/c/3JPyg8/QOgK) | 174 | Schizophrenia | Outpatient | Turkey | Europe & Central Asia | HIC | Cross-sectional | No | No | Low |
| Goldstein 2011 [(45)](https://paperpile.com/c/3JPyg8/yAOe) | 43093 | Bipolar disorders | Outpatient | US | North America | HIC | Cohort | No | No | Low |
| McElroy 2011 [(46)](https://paperpile.com/c/3JPyg8/J5IJ) | 875 | Bipolar disorders | Community | UK | Europe & Central Asia | HIC | Cross-sectional | No | No | Low |
| Mookhoek 2011 [(47)](https://paperpile.com/c/3JPyg8/X3PT) | 256 | Schizophrenia | Inpateint | The Netherlands | Europe & Central Asia | HIC | Cross-sectional | No | No | Low |
| Udo 2011 [(48)](https://paperpile.com/c/3JPyg8/ATTw) | 30 | Any | Inpateint | Ireland | Europe & Central Asia | HIC | Cross-sectional | No | No | High |
| Inamura 2012 [(49)](https://paperpile.com/c/3JPyg8/9F8k) | 15171 | Schizophrenia | Inpateint | Japan | East Asia & Pacific | HIC | Cross-sectional | No | Yes | Low |
| Centorrino 2012 [(50)](https://paperpile.com/c/3JPyg8/r673) | 76 | Schizophrenia | Inpateint | US | North America | HIC | Cross-sectional | No | No | Low |
| Centorrino 2012 [(50)](https://paperpile.com/c/3JPyg8/r673) | 173 | Bipolar disorders | Inpateint | US | North America | HIC | Cross-sectional | No | No | Low |
| Choong 2012 [(51)](https://paperpile.com/c/3JPyg8/YNi2) | 196 | Schizophrenia | Outpatient | Switzerland | Europe & Central Asia | HIC | Cross-sectional | No | Yes | Low |
| Feiler 2012 [(52)](https://paperpile.com/c/3JPyg8/LNNP) | 106 | Any | Inpateint | Australia | East Asia & Pacific | HIC | Cross-sectional | Yes | Yes | Low |
| Galletly 2012 [(53)](https://paperpile.com/c/3JPyg8/kFjb) | 1286 | Any | Community | Australia | East Asia & Pacific | HIC | Cross-sectional | No | Yes | Low |
| Gurpegui 2012 [(54)](https://paperpile.com/c/3JPyg8/v87x) | 648 | Bipolar disorders | Outpatient | Spain | Europe & Central Asia | HIC | Cross-sectional | No | No | Low |
| Gurpegui 2012 [(54)](https://paperpile.com/c/3JPyg8/v87x) | 648 | Schizophrenia | Outpatient | Spain | Europe & Central Asia | HIC | Cross-sectional | No | No | Low |
| Lan 2012 [(55)](https://paperpile.com/c/3JPyg8/ZEjd) | 1030 | Schizophrenia | Inpateint | Taiwan | East Asia & Pacific | LMIC | Cross-sectional | No | Yes | Low |
| Lee 2012 [(56)](https://paperpile.com/c/3JPyg8/EKwj) | 100 | Schizophrenia | Out and Inpatient | Singapore | East Asia & Pacific | HIC | Cross-sectional | Yes | Yes | Low |
| Schuster 2012 [(57)](https://paperpile.com/c/3JPyg8/EMxw) | 3714 | Schizophrenia | Outpatient | France | Europe & Central Asia | HIC | Propective Longitudinal | No | No | Medium |
| Janney 2014 [(58)](https://paperpile.com/c/3JPyg8/dj3z) | 60 | Bipolar disorders | Community | US | North America | HIC | Cross-sectional | No | Yes | Medium |
| Sugawara 2013 [(59)](https://paperpile.com/c/3JPyg8/x85U) | 225 | Schizophrenia | Outpatient | Japan | East Asia & Pacific | HIC | Cross-sectional | No | Yes | Low |
| Bener 2014 [(60)](https://paperpile.com/c/3JPyg8/bLlL) | 233 | Schizophrenia | Outpatient | Qatar | Middle East & North Africa | HIC | Cross-sectional | Yes | Yes | Medium |
| Gordan 2012 [(61)](https://paperpile.com/c/3JPyg8/iOKF) | 261 | Schizophrenia | Outpatient | Brazil | Latin America & Carribean | LMIC | Cross-sectional | No | Yes | High |
| Guo 2013 [(62)](https://paperpile.com/c/3JPyg8/bZlr) | 1108 | Schizophrenia | Outpatient | China | East Asia & Pacific | LMIC | Cross-sectional | No | No | Low |
| Minsky 2013 [(63)](https://paperpile.com/c/3JPyg8/C5sb) | 586 | Any | Out and Inpatient | US | North America | HIC | Cross-sectional | No | No | High |
| Nunes 2014 [(64)](https://paperpile.com/c/3JPyg8/1tLH) | 25 | Schizophrenia | Outpatient | Brazil | Latin America & Carribean | HIC | Cross-sectional | Yes | Yes | Low |
| Suzuki 2013 [(65)](https://paperpile.com/c/3JPyg8/TKVZ) | 171 | Schizophrenia | Inpateint | Japan | East Asia & Pacific | HIC | Cohort | No | No | Medium |
| Depp 2014 [(66)](https://paperpile.com/c/3JPyg8/4FBQ) | 358 | Schizophrenia | Outpatient | US | North America | HIC | Cross-sectional | No | No | Low |
| Depp 2014 [(66)](https://paperpile.com/c/3JPyg8/4FBQ) | 400 | Bipolar disorders | Outpatient | US | North America | HIC | Cross-sectional | No | No | Low |
| Foldemo 2014 [(67)](https://paperpile.com/c/3JPyg8/uMYY) | 903 | Schizophrenia | Outpatient | Sweden | Europe & Central Asia | HIC | Cross-sectional | Yes | Yes | Medium |
| Post 2014 [(68)](https://paperpile.com/c/3JPyg8/rJdM) | 165 | Bipolar disorders | Outpatient | Germany | Europe & Central Asia | HIC | Propective Longitudinal | No | No | Medium |
| Post 2014 [(68)](https://paperpile.com/c/3JPyg8/rJdM) | 166 | Bipolar disorders | Outpatient | The Netherlands | Europe & Central Asia | HIC | Propective Longitudinal | No | No | Medium |
| Post 2014 [(68)](https://paperpile.com/c/3JPyg8/rJdM) | 676 | Bipolar disorders | Outpatient | US | North America | HIC | Propective Longitudinal | No | No | Medium |
| Subramaniam 2014 [(69)](https://paperpile.com/c/3JPyg8/goc8) | 973 | Schizophrenia | Outpatient | Singapore | East Asia & Pacific | HIC | Cross-sectional | Yes | Yes | Low |
| Suzuki 2014 [(70)](https://paperpile.com/c/3JPyg8/AOFI) | 333 | Schizophrenia | Inpateint | Japan | East Asia & Pacific | HIC | Cross-sectional | Yes | Yes | Medium |
| Ventriglio 2014 [(71)](https://paperpile.com/c/3JPyg8/6CEE) | 66 | Any | Outpatient | Italy | Europe & Central Asia | HIC | Cross-sectional | No | No | Low |
| Bernstein 2015 [(72)](https://paperpile.com/c/3JPyg8/cHOE) | 62 | Bipolar disorders | Outpatient | US | North America | HIC | Cross-sectional | No | No | High |
| Marthoenis 2014 [(73)](https://paperpile.com/c/3JPyg8/itIJ) | 86 | Schizophrenia | Inpateint | Indonesia | East Asia & Pacific | LMIC | Cross-sectional | No | No | Medium |
| Sugai 2015 [(74)](https://paperpile.com/c/3JPyg8/YLrk) | 19678 | Schizophrenia | Out and Inpatient | Japan | East Asia & Pacific | HIC | Cross-sectional | No | No | Low |
| Bioque 2018 [(75)](https://paperpile.com/c/3JPyg8/KuoP) | 335 | Schizophrenia | Out and Inpatient | Spain | Europe & Central Asia | HIC | Cross-sectional | Yes | Yes | Low |
| Chouinard 2016 [(76)](https://paperpile.com/c/3JPyg8/yvk0) | 262 | Any | Inpateint | US | North America | HIC | Cross-sectional | No | No | Low |
| Lopuszanska 2016 [(77)](https://paperpile.com/c/3JPyg8/fXw3) | 91 | Any | Inpateint | Poland | Europe & Central Asia | HIC | Cross-sectional | No | Yes | Low |
| McElroy 2016 [(78)](https://paperpile.com/c/3JPyg8/jrJQ) | 1114 | Bipolar disorders | Community | UK | Europe & Central Asia | HIC | Cross-sectional | No | No | Medium |
| Annamalai 2017 [(79)](https://paperpile.com/c/3JPyg8/ZrKM) | 326 | Schizophrenia | Outpatient | US | North America | HIC | Cohort | Yes | Yes | Medium |
| Najar 2017 [(80)](https://paperpile.com/c/3JPyg8/NWzc) | 1141 | Any | Outpatient | Sweden | Europe & Central Asia | HIC | Cross-sectional | No | No | Medium |
| Oh 2017 [(81)](https://paperpile.com/c/3JPyg8/BHX8) | 167 | Schizophrenia | Inpateint | South Korea | East Asia & Pacific | HIC | Cross-sectional | No | No | Low |
| Susilova 2017 [(82)](https://paperpile.com/c/3JPyg8/ERGV) | 462 | Schizophrenia | Inpateint | Czech Republic | Europe & Central Asia | HIC | Propective Longitudinal | No | Yes | High |
| Berti 2018 [(83)](https://paperpile.com/c/3JPyg8/1YQq) | 193 | Schizophrenia | Outpatient | Italy | Europe & Central Asia | HIC | Cross-sectional | Yes | Yes | High |
| Blomqvist 2019 [(84)](https://paperpile.com/c/3JPyg8/rrDb) | 57 | Schizophreni | Outpatient | Sweden | Europe & Central Asia | HIC | Cross-sectional | No | No | High |
| Asaye 2018 [(85)](https://paperpile.com/c/3JPyg8/vZrL) | 360 | Any | Out and Inpatient | Ethiopia | Africa | LMIC | Cross-sectional | No | No | Low |
| Aziz 2018 [(86)](https://paperpile.com/c/3JPyg8/8p7Q) | 50 | Schizophrenia | Outpatient | Pakistan | South Asia | LMIC | Cross-sectional | No | No | High |
| Iruretagoyena 2018 [(87)](https://paperpile.com/c/3JPyg8/7ytJ) | 148 | Schizophrenia | Out and Inpatient | Chile | Latin America & Carribean | HIC | Cross-sectional | Yes | Yes | Low |
| Zhao 2018 [(88)](https://paperpile.com/c/3JPyg8/PXOZ) | 466 | Schizophrenia | Inpateint | China | East Asia & Pacific | LMIC | Cohort | Yes | Yes | Low |
| deCaluwe 2019 [(89)](https://paperpile.com/c/3JPyg8/bPzA) | 350 | Any | Out and Inpatient | Curaçao | Latin America & Carribean | HIC | Cross-sectional | No | No | Low |
| Tzeng 2020 [(90)](https://paperpile.com/c/3JPyg8/QSf5) | 260 | Any | Inpateint | Taiwan | East Asia & Pacific | LMIC | Cross-sectional | No | No | Medium |
| Vedal 2019 [(91)](https://paperpile.com/c/3JPyg8/MuXy) | 750 | Any | Outpatient | Norway | Europe & Central Asia | HIC | Cross-sectional | Yes | Yes | Low |
| Agaba 2020 [(92)](https://paperpile.com/c/3JPyg8/rxm2) | 304 | Any | Outpatient | Uganda | Africa | LMIC | Cross-sectional | No | No | Medium |
| Hammoudeh 2020 [(93)](https://paperpile.com/c/3JPyg8/sLNV) | 439 | Any | Out and Inpatient | Qatar | Middle East & North Africa | HIC | Cross-sectional | No | No | High |
| Park 2020 [(94)](https://paperpile.com/c/3JPyg8/X609) | 420 | Schizophrenia | Out and Inpatient | India | South Asia | LMIC | Cross-sectional | No | No | Low |
| Park 2020 [(94)](https://paperpile.com/c/3JPyg8/X609) | 463 | Schizophrenia | Out and Inpatient | Indonesia | East Asia & Pacific | LMIC | Cross-sectional | No | No | Low |
| Park 2020 [(94)](https://paperpile.com/c/3JPyg8/X609) | 145 | Schizophrenia | Out and Inpatient | Japan | East Asia & Pacific | HIC | Cross-sectional | No | No | Low |
| Park 2020 [(94)](https://paperpile.com/c/3JPyg8/X609) | 230 | Schizophrenia | Out and Inpatient | Malaysia | East Asia & Pacific | LMIC | Cross-sectional | No | No | Low |
| Park 2020 [(94)](https://paperpile.com/c/3JPyg8/X609) | 184 | Schizophrenia | Out and Inpatient | Taiwan | East Asia & Pacific | LMIC | Cross-sectional | No | No | Low |
| Smith 2016 [(95)](https://paperpile.com/c/3JPyg8/6OM7) | 46 | Any | Outpatient | UK | Europe & Central Asia | HIC | Cross-sectional | No | Yes | Low |
| Teshome 2020 [(96)](https://paperpile.com/c/3JPyg8/E4wL) | 245 | Any | Outpatient | Ethiopia | Africa | LMIC | Cross-sectional | No | No | Medium |
| Wang 2020 [(97)](https://paperpile.com/c/3JPyg8/YaiQ) | 325 | Schizophrenia | Community | China | East Asia & Pacific | LMIC | Cross-sectional | No | Yes | Low |
| Zavala 2020 [(98)](https://paperpile.com/c/3JPyg8/GCmq) | 1500 | Any | Out and Inpatient | Bangladesh | South Asia | LMIC | Cross-sectional | No | No | Low |
| Zavala 2020 [(98)](https://paperpile.com/c/3JPyg8/GCmq) | 858 | Any | Out and Inpatient | Pakistan | South Asia | LMIC | Cross-sectional | No | No | Low |

**Supplementary figure 1.** Funnel plot assessing publication bias

The visual inspection of the funnel plot from the selected studies shown no evidence of publication bias
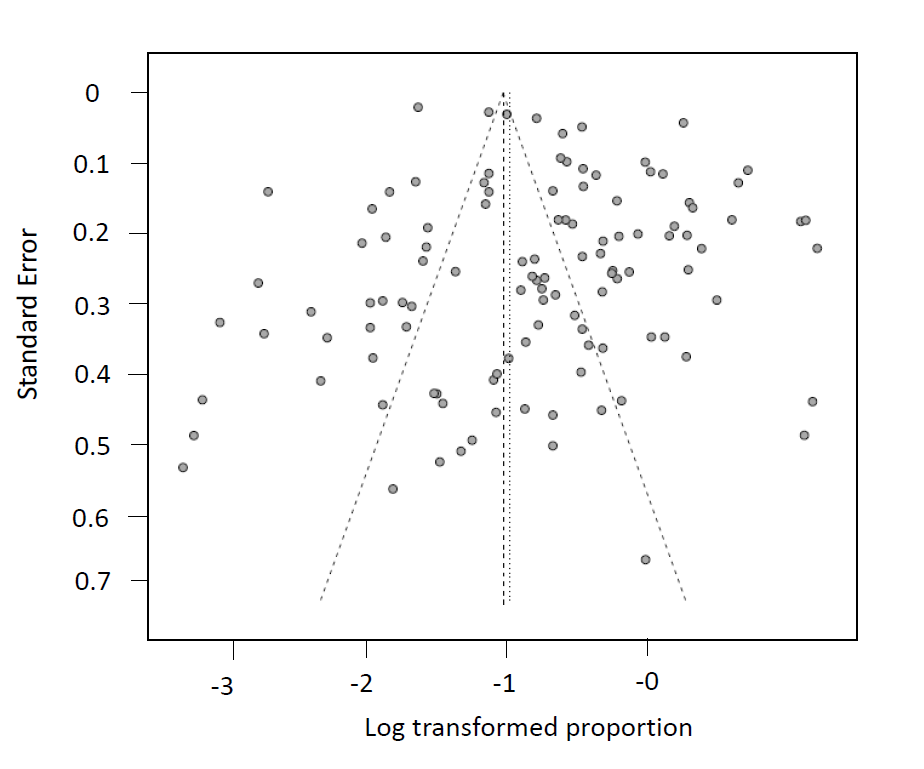


The visual inspection of the funnel plot from the selected studies shown no evidence of publication bias

**List of papers in a language other than English.**

1. Schizophrenia, obesity and pharmacotherapy-associated weight gain [(99)](https://paperpile.com/c/3JPyg8/7HSr).
2. The prevalence of metabolic syndrome and related factors in patients with schizophrenia [(100)](https://paperpile.com/c/3JPyg8/eMyg).
3. The prevalence of obesity and weight gain among the patients with psychotic disorder who use antipsychotic medication, one year follow-up [(101)](https://paperpile.com/c/3JPyg8/bkqM).
4. Metabolic side effects of atypical antipsychotic medication in outpatients of a University Hospital Clinic [(102)](https://paperpile.com/c/3JPyg8/TZYj).
5. Cardiovascular risk factors, cardiovascular risk and quality of life in patients with a severe mental disorder [(103)](https://paperpile.com/c/3JPyg8/qClK).
6. Dietary intake of young Icelanders with psychotic disorders and weight development over an 8-12 months period [(104)](https://paperpile.com/c/3JPyg8/jo6Q).
7. Survey of obesity and underweight among inpatients with schizophrenia in psychiatric hospitals throughout Japan[(105)](https://paperpile.com/c/3JPyg8/mwDm)
8. Prevalence of the metabolic syndrome among schizophrenic patients hospitalized in the Canary Islands [(106)](https://paperpile.com/c/3JPyg8/trom).
9. Disturbi mentali gravi e Sindrome Metabolica: meta-analisi e studio trasversale comparativo [(107)](https://paperpile.com/c/3JPyg8/EtbD).
10. Vigilance level for cardiovascular risk factors in schizophrenic patients [(108)](https://paperpile.com/c/3JPyg8/nVsU).
11. A study of risk factors for cardiovascular disease among schizophrenic patients using the rehabilitation mental health services at Kleppur [(109)](https://paperpile.com/c/3JPyg8/MsUo).
12. Risk factors for inactivity in patients in long-term care with severe mental illness [(110)](https://paperpile.com/c/3JPyg8/Uo3M).
13. Eating disorders in psychiatric patients during treatment with second generation antipsychotics [(111)](https://paperpile.com/c/3JPyg8/K1rD).
14. Body weight changes in chronic psychotic female in-patients [(112)](https://paperpile.com/c/3JPyg8/emf0).
15. Metabolic syndrome in bipolar affective disorder [(113)](https://paperpile.com/c/3JPyg8/PKFg).
16. Weight gain in patients with schizophrenia and schizoaffective disorder under antipsychotic treatment: A three-year naturalistic follow-up study [(114)](https://paperpile.com/c/3JPyg8/Ribf).

**References**

1. [Silverstone T, Smith G, Goodall E. Prevalence of obesity in patients receiving depot antipsychotics. Br J Psychiatry. 1988 Aug;153:214–7.](http://paperpile.com/b/3JPyg8/WFk4)

2. [Elmslie JL, Silverstone JT, Mann JI, Williams SM, Romans SE. Prevalence of overweight and obesity in bipolar patients. J Clin Psychiatry. 2000 Mar;61(3):179–84.](http://paperpile.com/b/3JPyg8/IbKa)

3. [Coodin S. Body mass index in persons with schizophrenia. Can J Psychiatry. 2001 Aug;46(6):549–55.](http://paperpile.com/b/3JPyg8/ax5V)

4. [Davidson S, Judd F, Jolley D, Hocking B, Thompson S, Hyland B. Cardiovascular risk factors for people with mental illness. Aust N Z J Psychiatry. 2001 Apr;35(2):196–202.](http://paperpile.com/b/3JPyg8/m6UI)

5. [Winkelman JW. Schizophrenia, obesity, and obstructive sleep apnea. J Clin Psychiatry. 2001 Jan;62(1):8–11.](http://paperpile.com/b/3JPyg8/W5VZ)

6. [Strassnig M, Brar JS, Ganguli R. Nutritional assessment of patients with schizophrenia: a preliminary study. Schizophr Bull. 2003;29(2):393–7.](http://paperpile.com/b/3JPyg8/LxbK)

7. [Daumit GL, Clark JM, Steinwachs DM, Graham CM, Lehman A, Ford DE. Prevalence and correlates of obesity in a community sample of individuals with severe and persistent mental illness. J Nerv Ment Dis. 2003 Dec;191(12):799–805.](http://paperpile.com/b/3JPyg8/aqlu)

8. [Hsiao C-C, Ree S-C, Chiang Y-L, Yeh S-S, Chen C-K. Obesity in schizophrenic outpatients receiving antipsychotics in Taiwan. Psychiatry Clin Neurosci. 2004 Aug;58(4):403–9.](http://paperpile.com/b/3JPyg8/GLSP)

9. [Strassnig M, Brar JS, Ganguli R. Self-reported body weight perception and dieting practices in community-dwelling patients with schizophrenia. Schizophr Res. 2005 Jun 15;75(2-3):425–32.](http://paperpile.com/b/3JPyg8/x7wX)

10. [Fagiolini A, Frank E, Scott JA, Turkin S, Kupfer DJ. Metabolic syndrome in bipolar disorder: findings from the Bipolar Disorder Center for Pennsylvanians. Bipolar Disord. 2005 Oct;7(5):424–30.](http://paperpile.com/b/3JPyg8/jlh6)

11. [Wang PW, Sachs GS, Zarate CA, Marangell LB, Calabrese JR, Goldberg JF, et al. Overweight and obesity in bipolar disorders. J Psychiatr Res. 2006 Dec;40(8):762–4.](http://paperpile.com/b/3JPyg8/cvvW)

12. [De Hert M, Peuskens B, van Winkel R, Kalnicka D, Hanssens L, Van Eyck D, et al. Body weight and self-esteem in patients with schizophrenia evaluated with B-WISE. Schizophr Res. 2006 Dec;88(1-3):222–6.](http://paperpile.com/b/3JPyg8/wBLd)

13. [Dickerson FB, Brown CH, Kreyenbuhl JA, Fang L, Goldberg RW, Wohlheiter K, et al. Obesity among individuals with serious mental illness. Acta Psychiatr Scand. 2006 Apr;113(4):306–13.](http://paperpile.com/b/3JPyg8/xla1)

14. [Faulkner G, Cohn T, Remington G, Irving H. Body mass index, waist circumference and quality of life in individuals with schizophrenia. Schizophr Res. 2007 Feb;90(1-3):174–8.](http://paperpile.com/b/3JPyg8/pS4H)

15. [Henderson DC, Borba CP, Daley TB, Boxill R, Nguyen DD, Culhane MA, et al. Dietary intake profile of patients with schizophrenia. Ann Clin Psychiatry. 2006 Apr;18(2):99–105.](http://paperpile.com/b/3JPyg8/WLQW)

16. [Kitabayashi Y, Narumoto J, Kitabayashi M, Fukui K. Body mass index among Japanese inpatients with schizophrenia. Int J Psychiatry Med. 2006;36(1):93–102.](http://paperpile.com/b/3JPyg8/KLPb)

17. [Leitão-Azevedo CL, Abreu MGB de, Guimarães LR, Moreno D, Lobato MI, Gama CS, et al. Overweight and obesity in schizophrenic patients taking clozapine compared to the use of other antipsychotics. Rev psiquiatr Rio Gd Sul. 2006 Aug;28(2):120–8.](http://paperpile.com/b/3JPyg8/Uh1h)

18. [Feeney L, Kelly F, Lee J, Kelly E, McLaughlin S, O’Boyle JH. Point prevalence of diabetes, obesity, hyperlipidaemia, hypertension and smoking in outpatients on clozapine. Ir J Psychol Med. 2007 Jun;24(2):59–61.](http://paperpile.com/b/3JPyg8/sVIc)

19. [Hellerstein DJ, Almeida G, Devlin MJ, Mendelsohn N, Helfand S, Dragatsi D, et al. Assessing obesity and other related health problems of mentally ill Hispanic patients in an urban outpatient setting. Psychiatr Q. 2007 Sep;78(3):171–81.](http://paperpile.com/b/3JPyg8/UdZY)

20. [Kim B, Kim S-J, Son J-I, Joo YH. Weight change in the acute treatment of bipolar I disorder: a naturalistic observational study of psychiatric inpatients. J Affect Disord. 2008 Jan;105(1-3):45–52.](http://paperpile.com/b/3JPyg8/CNUI)

21. [Mackin P, Bishop DR, Watkinson HMO. A prospective study of monitoring practices for metabolic disease in antipsychotic-treated community psychiatric patients. BMC Psychiatry. 2007 Jun 25;7:28.](http://paperpile.com/b/3JPyg8/5A5h)

22. [Nishiyama M, Komahashi T, Kimijima M, Ohrui M. Preliminary report of body mass index among people with and without schizophrenia in Japan. 2007; Available from:](http://paperpile.com/b/3JPyg8/vgrR) <https://dmu.repo.nii.ac.jp/?action=pages_view_main&active_action=repository_view_main_item_detail&item_id=552&item_no=1&page_id=28&block_id=52>

23. [Tirupati S, Chua L-E. Obesity and Metabolic Syndrome in a Psychiatric Rehabilitation Service [Internet]. Vol. 41, Australian & New Zealand Journal of Psychiatry. 2007. p. 606–10. Available from:](http://paperpile.com/b/3JPyg8/NRi2) <http://dx.doi.org/10.1080/00048670701392841>

24. [Maina G, Salvi V, Vitalucci A, D’Ambrosio V, Bogetto F. Prevalence and correlates of overweight in drug-naïve patients with bipolar disorder. J Affect Disord. 2008 Sep 1;110(1-2):149–55.](http://paperpile.com/b/3JPyg8/ndYc)

25. [Ainsah O, Salmi R, Osman CB, Shamsul AS. Relationships between antipsychotic medication and anthropometric measurements in patients with schizophrenia attending a psychiatric clinic in Malaysia. East Asian Arch Psychiatry. 2008 Mar;18:23+.](http://paperpile.com/b/3JPyg8/yWpy)

26. [Correll CU, Frederickson AM, Kane JM, Manu P. Equally increased risk for metabolic syndrome in patients with bipolar disorder and schizophrenia treated with second-generation antipsychotics. Bipolar Disord. 2008 Nov;10(7):788–97.](http://paperpile.com/b/3JPyg8/Etl1)

27. [Limosin F, Gasquet I, Leguay D, Azorin J-M, Rouillon F. Body mass index and prevalence of obesity in a French cohort of patients with schizophrenia. Acta Psychiatr Scand. 2008 Jul;118(1):19–25.](http://paperpile.com/b/3JPyg8/2FIv)

28. [McLaren L, Beck CA, Patten SB, Fick GH, Adair CE. The relationship between body mass index and mental health. A population-based study of the effects of the definition of mental health. Soc Psychiatry Psychiatr Epidemiol. 2008 Jan;43(1):63–71.](http://paperpile.com/b/3JPyg8/bdBM)

29. [van Winkel R, De Hert M, Van Eyck D, Hanssens L, Wampers M, Scheen A, et al. Prevalence of diabetes and the metabolic syndrome in a sample of patients with bipolar disorder. Bipolar Disord. 2008 Mar;10(2):342–8.](http://paperpile.com/b/3JPyg8/CXkz)

30. [Calkin C, van de Velde C, Růzicková M, Slaney C, Garnham J, Hajek T, et al. Can body mass index help predict outcome in patients with bipolar disorder? Bipolar Disord. 2009 Sep;11(6):650–6.](http://paperpile.com/b/3JPyg8/2WTk)

31. [Bell RC, Farmer S, Ries R, Srebnik D. Metabolic risk factors among medicaid outpatients with schizophrenia receiving second-generation antipsychotics. Psychiatr Serv. 2009 Dec;60(12):1686–9.](http://paperpile.com/b/3JPyg8/cDmN)

32. [Bernardo M, Cañas F, Banegas JR, Casademont J, Riesgo Y, Varela C, et al. Prevalence and awareness of cardiovascular risk factors in patients with schizophrenia: a cross-sectional study in a low cardiovascular disease risk geographical area. Eur Psychiatry. 2009 Oct;24(7):431–41.](http://paperpile.com/b/3JPyg8/9VVm)

33. [Bodén R, Haenni A, Lindström L, Sundström J. Biochemical risk factors for development of obesity in first-episode schizophrenia. Schizophr Res. 2009 Dec;115(2-3):141–5.](http://paperpile.com/b/3JPyg8/0Njp)

34. [de Almeida KM, de Macedo-Soares MB, Kluger Issler C, Antonio Amaral J, Caetano SC, da Silva Dias R, et al. Obesity and metabolic syndrome in Brazilian patients with bipolar disorder. Acta Neuropsychiatr. 2009 Apr;21(2):84–8.](http://paperpile.com/b/3JPyg8/xV1z)

35. [Fiedorowicz JG, Palagummi NM, Forman-Hoffman VL, Miller DD, Haynes WG. Elevated prevalence of obesity, metabolic syndrome, and cardiovascular risk factors in bipolar disorder. Ann Clin Psychiatry. 2008 Jul;20(3):131–7.](http://paperpile.com/b/3JPyg8/TQkj)

36. [Huang M-C, Lu M-L, Tsai C-J, Chen P-Y, Chiu C-C, Jian D-L, et al. Prevalence of metabolic syndrome among patients with schizophrenia or schizoaffective disorder in Taiwan. Acta Psychiatr Scand. 2009 Oct;120(4):274–80.](http://paperpile.com/b/3JPyg8/NJk8)

37. [Uma Devi P, Murugam S. Metabolic Disturbances in Schizophrenia Patients With Positive, Negative and Cognitive Symptoms. JK Science [Internet]. 2009;11(3). Available from:](http://paperpile.com/b/3JPyg8/nXRp) <http://www.jkscience.org/archive/vol113/3-Original%20Article%20-%20Metabolic%20Disturbances.pdf>

38. [Vancampfort D, Probst M, Sweers K, Maurissen K, Knapen J, De Hert M. Relationships between obesity, functional exercise capacity, physical activity participation and physical self-perception in people with schizophrenia. Acta Psychiatr Scand. 2011 Jun;123(6):423–30.](http://paperpile.com/b/3JPyg8/E3I2)

39. [Correll CU, Druss BG, Lombardo I, O’Gorman C, Harnett JP, Sanders KN, et al. Findings of a U.S. national cardiometabolic screening program among 10,084 psychiatric outpatients. Psychiatr Serv. 2010 Sep;61(9):892–8.](http://paperpile.com/b/3JPyg8/ErP7)

40. [Lee NY, Kim SH, Cho B, Lee YJ, Chang JS, Kang UG, et al. Patients taking medications for bipolar disorder are more prone to metabolic syndrome than Korea’s general population. Prog Neuropsychopharmacol Biol Psychiatry. 2010 Oct 1;34(7):1243–9.](http://paperpile.com/b/3JPyg8/zl2u)

41. [Mahendran R, Hendricks M, Chan YH. Weight gain in Asian patients on second-generation antipsychotics. Ann Acad Med Singapore. 2010 Feb;39(2):118–21.](http://paperpile.com/b/3JPyg8/brfa)

42. [Arango C, Bobes J, Kirkpatrick B, Garcia-Garcia M, Rejas J. Psychopathology, coronary heart disease and metabolic syndrome in schizophrenia spectrum patients with deficit versus non-deficit schizophrenia: findings from the CLAMORS study. Eur Neuropsychopharmacol. 2011 Dec;21(12):867–75.](http://paperpile.com/b/3JPyg8/rfEw)

43. [Chu K-Y, Yang N-P, Chou P, Chi L-Y, Chiu H-J. The relationship between body mass index, the use of second-generation antipsychotics, and dental caries among hospitalized patients with schizophrenia. Int J Psychiatry Med. 2011;41(4):343–53.](http://paperpile.com/b/3JPyg8/dCe9)

44. [Falissard B, Mauri M, Shaw K, Wetterling T, Doble A, Giudicelli A, et al. The METEOR study: frequency of metabolic disorders in patients with schizophrenia. Focus on first and second generation and level of risk of antipsychotic drugs. Int Clin Psychopharmacol. 2011 Nov;26(6):291–302.](http://paperpile.com/b/3JPyg8/QOgK)

45. [Goldstein BI, Liu S-M, Zivkovic N, Schaffer A, Chien L-C, Blanco C. The burden of obesity among adults with bipolar disorder in the United States. Bipolar Disord. 2011 Jun;13(4):387–95.](http://paperpile.com/b/3JPyg8/yAOe)

46. [McElroy SL, Frye MA, Hellemann G, Altshuler L, Leverich GS, Suppes T, et al. Prevalence and correlates of eating disorders in 875 patients with bipolar disorder. J Affect Disord. 2011 Feb;128(3):191–8.](http://paperpile.com/b/3JPyg8/J5IJ)

47. [Mookhoek EJ, de Vries WA, Hovens JEJM, Brouwers JRBJ, Loonen AJM. Risk factors for overweight and diabetes mellitus in residential psychiatric patients. Obes Facts. 2011 Oct 14;4(5):341–5.](http://paperpile.com/b/3JPyg8/X3PT)

48. [Udo I, Mooney M, Newman A. Prevalence of obesity and metabolic syndrome in a long-stay psychiatric unit. Ir J Psychol Med. 2011 Dec;28(4):205–8.](http://paperpile.com/b/3JPyg8/ATTw)

49. [Inamura Y, Sagae T, Nakamachi K, Murayama N. Body mass index of inpatients with schizophrenia in Japan. Int J Psychiatry Med. 2012;44(2):171–81.](http://paperpile.com/b/3JPyg8/9F8k)

50. [Centorrino F, Masters GA, Talamo A, Baldessarini RJ, Öngür D. Metabolic syndrome in psychiatrically hospitalized patients treated with antipsychotics and other psychotropics. Hum Psychopharmacol. 2012 Sep;27(5):521–6.](http://paperpile.com/b/3JPyg8/r673)

51. [Choong E, Bondolfi G, Etter M, Jermann F, Aubry J-M, Bartolomei J, et al. Psychotropic drug-induced weight gain and other metabolic complications in a Swiss psychiatric population. J Psychiatr Res. 2012 Apr;46(4):540–8.](http://paperpile.com/b/3JPyg8/YNi2)

52. [Feiler G, Chen RC-Y, Pantelis C, Lambert T. Health behaviours of community-treated patients with psychosis. Australas Psychiatry. 2012 Jun;20(3):208–13.](http://paperpile.com/b/3JPyg8/LNNP)

53. [Galletly CA, Foley DL, Waterreus A, Watts GF, Castle DJ, McGrath JJ, et al. Cardiometabolic risk factors in people with psychotic disorders: the second Australian national survey of psychosis. Aust N Z J Psychiatry. 2012 Aug;46(8):753–61.](http://paperpile.com/b/3JPyg8/kFjb)

54. [Gutiérrez-Rojas L, Gurpegui M, Martínez-Ortega JM, Jurado L. Overweight and obesity in patients with bipolar disorder or schizophrenia compared with a non-Psychiatric Sample [Internet]. Vol. 28, International Clinical Psychopharmacology. 2012. p. e63. Available from:](http://paperpile.com/b/3JPyg8/v87x) <http://dx.doi.org/10.1097/01.yic.0000423356.40365.10>

55. [Lan Y-L, Chen T-L. Prevalence of High Blood Pressure and its Relationship with Body Weight Factors among Inpatients with Schizophrenia in Taiwan. Asian Nurs Res . 2012 Mar;6(1):13–8.](http://paperpile.com/b/3JPyg8/ZEjd)

56. [Lee J, Nurjono M, Wong A, Salim A. Prevalence of metabolic syndrome among patients with schizophrenia in Singapore. Ann Acad Med Singapore. 2012 Oct;41(10):457–62.](http://paperpile.com/b/3JPyg8/EKwj)

57. [Schuster J-P, Raucher-Chéné D, Lemogne C, Rouillon F, Gasquet I, Leguay D, et al. Impact of switching or initiating antipsychotic treatment on body weight during a 6-month follow-up in a cohort of patients with schizophrenia. J Clin Psychopharmacol. 2012 Oct;32(5):672–7.](http://paperpile.com/b/3JPyg8/EMxw)

58. [Janney CA, Fagiolini A, Swartz HA, Jakicic JM, Holleman RG, Richardson CR. Are adults with bipolar disorder active? Objectively measured physical activity and sedentary behavior using accelerometry. J Affect Disord. 2014 Jan;152-154:498–504.](http://paperpile.com/b/3JPyg8/dj3z)

59. [Sugawara N, Yasui-Furukori N, Sato Y, Saito M, Furukori H, Nakagami T, et al. Body mass index and quality of life among outpatients with schizophrenia in Japan. BMC Psychiatry. 2013 Apr 9;13:108.](http://paperpile.com/b/3JPyg8/x85U)

60. [Bener A, Al-Hamaq AOA, Dafeeah EE. A two fold risk of metabolic syndrome in a sample of patients with schizophrenia: Do consanguinity and family history increase risk? [Internet]. Vol. 8, Diabetes & Metabolic Syndrome: Clinical Research & Reviews. 2014. p. 24–9. Available from:](http://paperpile.com/b/3JPyg8/bLlL) <http://dx.doi.org/10.1016/j.dsx.2013.10.003>

61. [Gordon P, Louza MR, Xavier. Weight gain, metabolic disturbances, and physical health care in a Brazilian sample of outpatients with schizophrenia [Internet]. Neuropsychiatric Disease and Treatment. 2013. p. 133. Available from:](http://paperpile.com/b/3JPyg8/iOKF) <http://dx.doi.org/10.2147/ndt.s37019>

62. [Guo X, Zhang Z, Zhai J, Wu R, Liu F, Zhao J, et al. The relationship between obesity and health-related quality of life in Chinese patients with schizophrenia. Int J Psychiatry Clin Pract. 2013 Feb;17(1):16–20.](http://paperpile.com/b/3JPyg8/bZlr)

63. [Minsky S, Vreeland B, Miller M, Gara M. Concordance between measured and self-perceived weight status of persons with serious mental illness. Psychiatr Serv. 2013 Jan;64(1):91–3.](http://paperpile.com/b/3JPyg8/C5sb)

64. [Nunes D, Eskinazi B, Rockett FC, Delgado VB, Perry IDS. Nutritional status, food intake and cardiovascular disease risk in individuals with schizophrenia in southern Brazil: A case–control study [Internet]. Vol. 7, Revista de Psiquiatría y Salud Mental (English Edition). 2014. p. 72–9. Available from:](http://paperpile.com/b/3JPyg8/1tLH) <http://dx.doi.org/10.1016/j.rpsmen.2014.01.001>

65. [Suzuki Y, Mikami T, Tajiri M, Kunizuka T, Abe H, Someya T. Effects of Hospitalization in a Psychiatric Ward on the Body Weight of Japanese Patients with Schizophrenia [Internet]. Vol. 45, The International Journal of Psychiatry in Medicine. 2013. p. 261–8. Available from:](http://paperpile.com/b/3JPyg8/TKVZ) <http://dx.doi.org/10.2190/pm.45.3.e>

66. [Depp CA, Strassnig M, Mausbach BT, Bowie CR, Wolyniec P, Thornquist MH, et al. Association of obesity and treated hypertension and diabetes with cognitive ability in bipolar disorder and schizophrenia. Bipolar Disord. 2014 Jun;16(4):422–31.](http://paperpile.com/b/3JPyg8/4FBQ)

67. [Foldemo A, Wärdig R, Bachrach-Lindström M, Edman G, Holmberg T, Lindström T, et al. Health-related quality of life and metabolic risk in patients with psychosis [Internet]. Vol. 152, Schizophrenia Research. 2014. p. 295–9. Available from:](http://paperpile.com/b/3JPyg8/uMYY) <http://dx.doi.org/10.1016/j.schres.2013.11.029>

68. [Post RM, Altshuler LL, Leverich GS, Frye MA, Suppes T, McElroy SL, et al. More medical comorbidities in patients with bipolar disorder from the United States than from the Netherlands and Germany. J Nerv Ment Dis. 2014 Apr;202(4):265–70.](http://paperpile.com/b/3JPyg8/rJdM)

69. [Subramaniam M, Lam M, Guo ME, He VYF, Lee J, Verma S, et al. Body mass index, obesity, and psychopathology in patients with schizophrenia. J Clin Psychopharmacol. 2014 Feb;34(1):40–6.](http://paperpile.com/b/3JPyg8/goc8)

70. [Suzuki Y, Sugai T, Fukui N, Watanabe J, Ono S, Tsuneyama N, et al. High prevalence of underweight and undernutrition in Japanese inpatients with schizophrenia. Psychiatry Clin Neurosci. 2014 Jan;68(1):78–82.](http://paperpile.com/b/3JPyg8/AOFI)

71. [Ventriglio A, Gentile A, Baldessarini RJ, Martone S, Vitrani G, La Marca A, et al. Improvements in metabolic abnormalities among overweight schizophrenia and bipolar disorder patients. Eur Psychiatry. 2014 Sep;29(7):402–7.](http://paperpile.com/b/3JPyg8/6CEE)

72. [Bernstein EE, Nierenberg AA, Deckersbach T, Sylvia LG. Eating behavior and obesity in bipolar disorder. Aust N Z J Psychiatry. 2015 Jun;49(6):566–72.](http://paperpile.com/b/3JPyg8/cHOE)

73. [Marthoenis M, Aichberger M, Puteh I, Schouler-Ocak M. Low rate of obesity among psychiatric inpatients in Indonesia. Int J Psychiatry Med. 2014;48(3):175–83.](http://paperpile.com/b/3JPyg8/itIJ)

74. [Sugai T, Suzuki Y, Yamazaki M, Shimoda K, Mori T, Ozeki Y, et al. High prevalence of underweight and undernutrition in Japanese inpatients with schizophrenia: a nationwide survey. BMJ Open. 2015 Dec 9;5(12):e008720.](http://paperpile.com/b/3JPyg8/YLrk)

75. [Bioque M, a Paz García-Portilla M, García-Rizo C, Cabrera B, Lobo A, González-Pinto A, et al. Evolution of metabolic risk factors over a two-year period in a cohort of first episodes of psychosis [Internet]. Vol. 193, Schizophrenia Research. 2018. p. 188–96. Available from:](http://paperpile.com/b/3JPyg8/KuoP) <http://dx.doi.org/10.1016/j.schres.2017.06.032>

76. [Chouinard V-A, Pingali SM, Chouinard G, Henderson DC, Mallya SG, Cypess AM, et al. Factors associated with overweight and obesity in schizophrenia, schizoaffective and bipolar disorders. Psychiatry Res. 2016 Mar 30;237:304–10.](http://paperpile.com/b/3JPyg8/yvk0)

77. [Łopuszańska U, Skórzyńska-Dziduszko K, Prendecka M, Makara-Studzińska M. Overweight, obesity and cognitive functions disorders in group of people suffering from mental illness. Psychiatr Pol. 2016;50(2):393–406.](http://paperpile.com/b/3JPyg8/fXw3)

78. [McElroy SL, Crow S, Blom TJ, Cuellar-Barboza AB, Prieto ML, Veldic M, et al. Clinical features of bipolar spectrum with binge eating behaviour. J Affect Disord. 2016 Sep 1;201:95–8.](http://paperpile.com/b/3JPyg8/jrJQ)

79. [Annamalai A, Kosir U, Tek C. Prevalence of obesity and diabetes in patients with schizophrenia. World J Diabetes. 2017 Aug 15;8(8):390–6.](http://paperpile.com/b/3JPyg8/ZrKM)

80. [Najar H, Joas E, Kardell M, Pålsson E, Landén M. Weight gain with add-on second-generation antipsychotics in bipolar disorder: a naturalistic study. Acta Psychiatr Scand. 2017 Jun;135(6):606–11.](http://paperpile.com/b/3JPyg8/NWzc)

81. [Oh E, Song E, Shin J. Individual Factors Affecting Self-esteem, and Relationships Among Self-esteem, Body Mass Index, and Body Image in Patients With Schizophrenia. Arch Psychiatr Nurs. 2017 Dec;31(6):588–95.](http://paperpile.com/b/3JPyg8/BHX8)

82. [Sušilová L, Češková E, Hampel D, Sušil A, Šimůnek J. Changes in BMI in hospitalized patients during treatment with antipsychotics, depending on gender and other factors. Int J Psychiatry Clin Pract. 2017 Jun;21(2):112–7.](http://paperpile.com/b/3JPyg8/ERGV)

83. [Berti L, Bonfioli E, Chioffi L, Morgante S, Mazzi MA, Burti L. Lifestyles of Patients with Functional Psychosis Compared to Those of a Sample of the Regional General Population: Findings from a Study in a Community Mental Health Service of the Veneto Region, Italy [Internet]. Vol. 54, Community Mental Health Journal. 2018. p. 1050–6. Available from:](http://paperpile.com/b/3JPyg8/1YQq) <http://dx.doi.org/10.1007/s10597-017-0223-7>

84. [Blomqvist M, Ivarsson A, Carlsson I-M, Sandgren A, Jormfeldt H. Health Risks among People with Severe Mental Illness in Psychiatric Outpatient Settings. Issues Ment Health Nurs. 2018 Jul;39(7):585–91.](http://paperpile.com/b/3JPyg8/rrDb)

85. [Asaye S, Bekele S, Tolessa D, Cheneke W. Metabolic syndrome and associated factors among psychiatric patients in Jimma University Specialized Hospital, South West Ethiopia. Diabetes Metab Syndr. 2018 Sep;12(5):753–60.](http://paperpile.com/b/3JPyg8/vZrL)

86. [Aziz R, Haq AU, Butt BM. Screening for Metabolic Syndrome in Patients on Antipsychotic Medication-A Cross-Sectional Study in a Pakistani Sample. Age. 2018;35:10–9.](http://paperpile.com/b/3JPyg8/8p7Q)

87. [Iruretagoyena B, Castañeda CP, Undurraga J, Nachar R, Mena C, Gallardo C, et al. High prevalence of metabolic alterations in Latin American patients at initial stages of psychosis. Early Interv Psychiatry. 2019 Dec;13(6):1382–8.](http://paperpile.com/b/3JPyg8/7ytJ)

88. [Zhao S, Xia H, Mu J, Wang L, Zhu L, Wang A, et al. 10-year CVD risk in Han Chinese mainland patients with schizophrenia [Internet]. Vol. 264, Psychiatry Research. 2018. p. 322–6. Available from:](http://paperpile.com/b/3JPyg8/PXOZ) <http://dx.doi.org/10.1016/j.psychres.2018.04.020>

89. [Caluwé L de, de Caluwé L, van Buitenen N, Gelan PJ, Crunelle CL, Thomas R, et al. Prevalence of metabolic syndrome and its associated risk factors in an African–Caribbean population with severe mental illness [Internet]. Vol. 281, Psychiatry Research. 2019. p. 112558. Available from:](http://paperpile.com/b/3JPyg8/bPzA) <http://dx.doi.org/10.1016/j.psychres.2019.112558>

90. [Tzeng W-C, Chiang Y-S, Feng H-P, Chien W-C, Tai Y-M, Chen M-J. Gender differences in metabolic syndrome risk factors among patients with serious mental illness. Int J Ment Health Nurs. 2020 Apr;29(2):254–65.](http://paperpile.com/b/3JPyg8/QSf5)

91. [Vedal TSJ, Steen NE, Birkeland KI, Dieset I, Reponen EJ, Laskemoen JF, et al. Adipokine levels are associated with insulin resistance in antipsychotics users independently of BMI. Psychoneuroendocrinology. 2019 May;103:87–95.](http://paperpile.com/b/3JPyg8/MuXy)

92. [Agaba DC, Migisha R, Katamba G, Ashaba S. Cardio-metabolic abnormalities among patients with severe mental illness at a Regional Referral Hospital in southwestern Uganda [Internet]. Vol. 15, PLOS ONE. 2020. p. e0235956. Available from:](http://paperpile.com/b/3JPyg8/rxm2) <http://dx.doi.org/10.1371/journal.pone.0235956>

93. [Hammoudeh S, Al Lawati H, Ghuloum S, Iram H, Yehya A, Becetti I, et al. Risk Factors of Metabolic Syndrome Among Patients Receiving Antipsychotics: A Retrospective Study. Community Ment Health J. 2020 May;56(4):760–70.](http://paperpile.com/b/3JPyg8/sLNV)

94. [Park S-C, Tripathi A, Avasthi A, Grover S, Tanra AJ, Kato TA, et al. Relationship Between Body Mass Index and Extrapyramidal Symptoms in Asian Patients with Schizophrenia: The Research on Asian Psychotropic Prescription Patterns for Antipsychotics (REAP-AP). Psychiatr Danub. 2020 Summer;32(2):176–86.](http://paperpile.com/b/3JPyg8/X609)

95. [Smith J, Griffiths LA, Band M, Horne D. Cardiometabolic Risk in First Episode Psychosis Patients. Front Endocrinol . 2020 Nov 24;11:564240.](http://paperpile.com/b/3JPyg8/6OM7)

96. [Teshome T, Kassa DH, Hirigo AT. Prevalence and Associated Factors of Metabolic Syndrome Among Patients with Severe Mental Illness at Hawassa, Southern-Ethiopia. Diabetes Metab Syndr Obes. 2020 Feb 27;13:569–79.](http://paperpile.com/b/3JPyg8/E4wL)

97. [Wang J, Zhang Y, Yang Y, Liu Z, Xia L, Li W, et al. The prevalence and independent influencing factors of obesity and underweight in patients with schizophrenia: a multicentre cross-sectional study. Eat Weight Disord. 2021 Jun;26(5):1365–74.](http://paperpile.com/b/3JPyg8/YaiQ)

98. [Zavala GA, Haidar AC, Prasad-Muliyala K, Aslam F, Huque R, Khalid H, et al. The Physical Health of People With Severe Mental Illness in South Asia: A Cross-Sectional Multi-Country Survey [Internet]. SSRN Electronic Journal. Available from:](http://paperpile.com/b/3JPyg8/GCmq) <http://dx.doi.org/10.2139/ssrn.3736709>

99. [Carpiniello B, Corda E, Maccioni R, Pinna F. Schizophrenia, obesity and pharmacotherapy-associated weight gain. Clin Ter. 2008 Sep;159(5):299–306.](http://paperpile.com/b/3JPyg8/7HSr)

100. [Cerit C, Özten E, Yildiz M. The Prevalence of Metabolic Syndrome and Related Factors in Patients With Schizophrenia. Turk Psikiyatri Derg [Internet]. 2008;19(2). Available from:](http://paperpile.com/b/3JPyg8/eMyg) <http://www.turkpsikiyatri.com/Data/UnpublishedArticles/en/metabolicSyndrome.pdf>

101. [Cerit C, Yildiz M, Candan S. The prevalence of obesity and weight gain among the patients with psychotic disorder who use antipsychotic medication, one year follow-up. KLINIK PSIKOFARMAKOLOJI BULTENI. 2006;16(4):233.](http://paperpile.com/b/3JPyg8/bkqM)

102. [Cumurcu BE, Tümüklü MN, Çelikel FÇ, Demir S, Others. Metabolic side effects of atypical antipsychotic medication in outpatients of a University Hospital Clinic [Bir Üniversite Hastânesinde psikotik bozukluk hastalarinda atipik antipsikotik kullaniminin metabolik etkileri]. 2009; Available from:](http://paperpile.com/b/3JPyg8/TZYj) <http://earsiv.gop.edu.tr/xmlui/handle/20.500.12881/8533>

103. [Foguet Boreu Q, Roura Poch P, Bullón Chia A, Mauri Martin C, Gordo Serra N, Cecília Costa R, et al. Cardiovascular risk factors, cardiovascular risk and quality of life in patients with a severe mental disorder. Aten Primaria. 2013 Mar;45(3):141–8.](http://paperpile.com/b/3JPyg8/qClK)

104. [Fridthjofsdottir HG, Geirsdottir OG, Jonsdottir H, Steingrimsdottir L, Thorsdottir I, Thorgeirsdottir H, et al. [Dietary intake of young Icelanders with psychotic disorders and weight development over an 8-12 months period]. Laeknabladid. 2017 Jun;103(6):281–6.](http://paperpile.com/b/3JPyg8/jo6Q)

105. [Inamura Y, Sagae T, Kushida O, Nakamachi K, Murayama N. Survey of obesity and underweight among inpatients with schizophrenia in psychiatric hospitals throughout Japan. Seishin Shinkeigaku Zasshi. 2013;115(1):10–21.](http://paperpile.com/b/3JPyg8/mwDm)

106. [Sánchez-Araña Moreno T, Touriño González R, Hernández Fleta JL, León Pérez P. Prevalence of the metabolic syndrome among schizophrenic patients hospitalized in the Canary Islands. Actas Esp Psiquiatr. 2007 Nov;35(6):359–67.](http://paperpile.com/b/3JPyg8/trom)

107. [Bartoli DF. Disturbi mentali gravi e Sindrome Metabolica: meta-analisi e studio trasversale comparativo. Available from:](http://paperpile.com/b/3JPyg8/EtbD) <https://boa.unimib.it/bitstream/10281/42833/1/phd_unimib_734457.pdf>

108. [Rouillon F, Van Ganse E, Vekhoff P, Arnaud R, Depret-Bixio L, Dillenschneider A. Niveau de vigilance des psychiatres pour les facteurs de risque cardiovasculaire chez les patients schizophrènes. Encephale. 2015 Feb;41(1):70–7.](http://paperpile.com/b/3JPyg8/nVsU)

109. [Sveinsson O, Thorleifsson K, Aspelund T, Kolbeinsson H. A study of risk factors for cardiovascular disease among schizophrenic patients using the rehabilitation mental health services at Kleppur. Laeknabladid. 2012 Jul;98(7-8):399–402.](http://paperpile.com/b/3JPyg8/MsUo)

110. [Tenback DE, van Kessel F, Jessurun J, Pijl YJ, Heerdink ER, van Harten PN. Risk factors for inactivity in patients in long-term care with severe mental illness. Tijdschr Psychiatr. 2013;55(2):83–91.](http://paperpile.com/b/3JPyg8/Uo3M)

111. [Vasilenko LM, Gorobets LN, Bulanov VS, Litvinov AV, Ivanova GP, Tsarenko MA, et al. [Eating disorders in psychiatric patients during treatment with second generation antipsychotics]. Zh Nevrol Psikhiatr Im S S Korsakova. 2015;115(7):60–7.](http://paperpile.com/b/3JPyg8/K1rD)

112. [Yang P-C, Jou J-F, Wang C-W, Chong M-Y. Body Weight Changes in Chronice Psychotic Female In-Patients. 中華民國營養學會雜誌. 1997 Nov 1;22(4):457–64.](http://paperpile.com/b/3JPyg8/emf0)

113. [Yldz B, Turan MT, Beirli A. Metabolic Syndrome in Bipolar Affective Disorder. ERCYES MEDICAL JOURNAL. 33(3):197–204.](http://paperpile.com/b/3JPyg8/PKFg)

114. [Yildiz M. Weight gain in patients with schizophrenia and schizoaffective disorder under antipsychotic treatment: A three-year naturalistic follow-up study. Yildiz M, “Weight gain in patients with schizophrenia and schizoaffective disorder under antipsychotic treatment: A three-year naturalistic follow-up study”, KLINIK PSIKOFARMAKOLOJI BULTENI-BULLETIN OF CLINICAL PSYCHOPHARMACOLOGY, cilt [Internet]. 2008 [cited 2021 Aug 27];18. Available from:](http://paperpile.com/b/3JPyg8/Ribf) <https://avesis.kocaeli.edu.tr/yayin/f3582b6f-814c-4d5d-a178-f9fcb9d25573/weight-gain-in-patients-with-schizophrenia-and-schizoaffective-disorder-under-antipsychotic-treatment-a-three-year-naturalistic-follow-up-study>
